# An Artificial Intelligence‐Based Computer Vision Model for Human Sperm Concentration, Motility, and Kinematics Analysis

**DOI:** 10.1002/smmd.70026

**Published:** 2026-01-09

**Authors:** Sahar Shahali, David Mortimer, Moira K. O'Bryan, Robert McLachlan, Deirdre Zander‐Fox, Klaus Ackermann, Gulfam Ahmad, Adrian Neild, Reza Nosrati

**Affiliations:** ^1^ Department of Mechanical and Aerospace Engineering Monash University Clayton Australia; ^2^ Oozoa Biomedical Inc West Vancouver British Columbia Canada; ^3^ School of BioSciences Bio21 Molecular Science and Biotechnology Institute University of Melbourne Parkville Australia; ^4^ Monash IVF Group Cremorne Australia; ^5^ Clinical Andrology Hudson Institute of Medical Research Monash University Clayton Australia; ^6^ Biomedicine Discovery Institute Monash University Clayton Australia; ^7^ School of Biomedicine University of Adelaide Adelaide Australia; ^8^ SoDa Labs and Department of Econometrics and Business Statistics Monash Business School Clayton Australia; ^9^ Andrology Royal Children's Hospital Melbourne Australia

**Keywords:** andrology, artificial intelligence, computer vision, semen analysis, sperm motility

## Abstract

Accurate assessment of sperm concentration and motility is critical for the diagnosis and management of male infertility. However, current methods, manual hemocytometer counting and commercial computer‐aided sperm analysis (CASA) systems, are limited by labor intensity, human error, and variable performance under diverse sample conditions. Here, we present an artificial intelligence (AI)‐driven computer vision tool for high‐resolution, quantitative analysis of sperm motility and concentration. In a prospective study of 26 semen samples (22 patients, 4 donors), we benchmarked the AI model against manual tracking (using Fiji software) and a commercial CASA system (Hamilton Thorne IVOS II). Our method computed concentration and motility parameters, including straight‐line velocity (VSL), curvilinear velocity (VCL), average path velocity (VAP), linearity (LIN), amplitude of lateral head displacement (ALH_max_), and beat cross frequency (BCF). Calibration using donor samples enabled accurate mapping of tracked sperm counts to concentrations. The AI tool presented a strong linear correlation with manual tracking (*R* ^2^ = 0.93–0.98; Root Mean Square Error (RMSE) = 3.3–7.3 μm/s for VSL, VCL, VAP), and outperformed CASA in both accuracy and consistency across all motility parameters. Post‐calibration, ALH_max_ and BCF estimates improved substantially, with a 30%–50% reduction in RMSE. Grading of sperm motility by the AI model aligned closely with manual classification, avoiding the systematic misclassification typically observed with CASA. Furthermore, the AI system exhibited higher repeatability and robustness across duplicate samples and variable imaging conditions, with deviations below ± 2%. These findings demonstrate that our AI‐based tool offers a quantitative and reliable alternative to current semen analysis platforms, supporting improved fertility diagnostics and potentially a more informative treatment process.

## Key Points

- We developed an AI‐driven computer vision model for automated analysis of human sperm concentration, motility, and kinematics.
- The model was benchmarked against both manual tracking and a commercial CASA system, demonstrating strong linear correlation (*R* ^2^ = 0.93–0.98) with manual results.
- Our calibrated AI model outperformed CASA in accuracy and repeatability for motility parameters such as VSL, VCL, VAP, and BCF.
- The calibrated AI model achieved high robustness to changes in imaging conditions and maintained consistency in repeated assessments.
- This AI‐based system provides a reliable, reproducible, and accessible alternative for clinical sperm analysis in fertility diagnostics.

## Graphical abstract

A computer‐vision method measures sperm concentration and motility from recorded videos, benchmarked and calibrated against manual tracking to ensure consistency. It can potentially support routine fertility assessments with high accuracy.

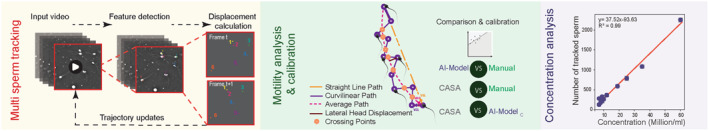

## 1 Introduction

Infertility affects an estimated 15% of couples worldwide, with male infertility contributing to nearly half of all infertility cases [1, 2, 3, 4]. Among the key parameters used to assess male fertility, sperm motility plays a central role [5, 6, 7, 8] not only as a predictor of sperm function and fertilization success [9, 10, 11] but also as a determinant of treatment strategy [12, 13], embryo quality [14], and clinical outcomes [15, 16]. Higher sperm motility levels are associated with increased fertilization and pregnancy rates [17], while also reducing the need for invasive infertility treatment procedures such as intracytoplasmic sperm injection (ICSI). Therefore, accurate and reproducible assessment of sperm motility is vital for both infertility diagnosis and treatment planning in reproductive medicine.

Current semen analysis methods in clinical andrology laboratories rely heavily on the evaluation of sperm concentration and motility [18]. Traditionally, concentration is measured using hemocytometers with improved Neubauer ruling, as recommended by the World Health Organization WHO 6th ed. (WHO6) [19]. While this method remains the gold standard, it is labor‐intensive, time‐consuming, and susceptible to human error [20, 21], particularly in pipetting, dilution, and visualization steps [22, 23, 24] that introduce variability affecting reproducibility and interpretation. In addition to qualitative visual inspection by light microscopy, sperm motility is frequently assessed using CASA systems [25, 26, 27]. CASA combines digital microscopy, video capture at 50–60 frames per second (fps), and image processing algorithms for automated quantitative assessments. While CASA could in theory improve standardization and throughput, accuracy is compromised by sperm aggregation, background debris, and imaging artifacts [28, 29, 30]. Moreover, CASA systems show reduced accuracy outside certain concentration ranges [22], with the CASA manual specifically noting that tracking errors become increasingly significant above 30 million/mL (M/mL) due to sperm overlap and collisions during acquisition, thus necessitating dilution and repeated assessments. Hence, CASA system performance is often restricted to specific equipment and settings, making it costly and reducing flexibility in diverse laboratory environments. Importantly, most CASA platforms deliver only population‐level kinematics, limiting their usefulness for single‐cell procedures such as ICSI. These systems have not yet been rigorously validated or benchmarked against ground‐truth methods to achieve the necessary accuracy and precision [30]. These limitations underscore the need for robust, cost‐effective, and adaptable tools that deliver accurate and reproducible concentration and motility measurements in routine practice.

Artificial intelligence (AI) and computer vision technologies have emerged as powerful alternatives for high‐throughput single‐cell tracking and analysis [31, 32, 33, 34], offering enhanced automation and accuracy. A few AI‐based models have been proposed to detect and track sperm movement [35]. Early approaches, such as the Joint Probabilistic Data Association Filter (JPDAF), allowed for population‐level motility estimation but lacked detailed kinematic profiling at the single‐cell level [36]. Subsequent advancements have integrated deep learning to improve detection and multi‐object tracking. Mohammadi et al. [37] employed RetinaNet with a ResNet50 backbone and feature pyramid networks to enhance multi‐scale sperm detection, combined with a modified CSR‐DCF tracker, achieving a high tracking performance with an F1‐score of 96.6%. Hidayatullah et al. [38] introduced a Tracking‐Grid framework for bull sperm, which improved detection under occlusion and motion blur, achieving 73.2% tracking accuracy. Complementary to these, MotilitAI [39] applied unsupervised tracking and regression models to predict sperm motility grades from videos. Later, studies focused on CASA‐derived metrics and morphological clustering to infer fertility traits and population‐level motility behavior [40, 41, 42]. Valiuškaitė et al. [43] applied a Faster R‐CNN to classify sperm motility based on WHO6 categories, with downstream heuristic algorithms for estimating motility grades. Somasundaram et al. [44] further improved segmentation, achieving an accuracy of 97.37%, by integrating an elliptic scanning and tail‐head motion algorithm. To address technical challenges in automated sperm tracking, particularly nonspecific sperm aggregation and overlapping trajectories, Zhu et al. [45] developed a two‐stage algorithm combining grid‐based modeling with graph theory for precise sperm head segmentation. This method significantly enhanced tracking accuracy by reducing false positives arising from densely clustered cells. More advanced AI models, such as multilayer Long Short‐Term Memory (LSTM) networks, have also been introduced to predict sperm swimming trajectories, achieving a mean location error of 4–8 μm [46]. Despite this progress, most of these AI models rely on training data, are limited to specific imaging conditions, require a high‐performance workstation, and lack validation against a benchmarked method. Accordingly, rigorous validation of reliability, accuracy, and clinical applicability is still lacking, preventing routine integration into andrology laboratories.

Here, we present a computer vision tool for automated sperm concentration and motility analysis. Our system was benchmarked against manual tracking using Fiji (an open‐source image analysis software) and the commercial CASA IVOS II system, evaluating motility parameters across 26 donor and patient samples. We further calibrate the model to correct systematic biases in selected motility parameters and evaluate its repeatability and robustness to changes in imaging conditions. This tool offers an alternative to commercial CASA systems for evaluating sperm concentration and motility, as it can be implemented using standard laboratory imaging setups, making it highly accessible for both diagnostic and treatment applications.

## 2 Materials and Methods

### 2.1 Study Population and Sample Collection

A total of 26 human semen samples were used in this study, comprising 4 donor samples obtained from Monash University and 22 patient samples collected from the Andrology Laboratory at the Royal Children's Hospital, Melbourne. Statistical analysis was performed using the Exact test, and a *p*‐value lower than 0.05 was considered significant. This study was approved by the Monash University Human Research Ethics Committee (Project ID 26713). The donor samples were specifically used to establish the calibration curve for sperm concentration analysis, while the patient samples were used to evaluate sperm motility and test the model's performance in sperm concentration analysis. All participants provided written informed consent, and samples were collected after 2–8 days of sexual abstinence, following standard clinical protocols. For all experiments, fresh semen samples were collected in sterile containers and allowed to liquefy at 37°C for 30 min before analysis. A total of 22 patient samples were analyzed, including eight undiluted samples, 12 samples diluted at a 1‐in‐5 ratio, and two samples diluted at a 1‐in‐2 ratio. Dilutions were performed according to the Andrology laboratory's standard diagnostic protocol, based on the initial concentration of each sample. Specifically, samples with sperm concentrations exceeding the optimal CASA range analysis were diluted to ensure accurate and reliable measurements. To cover a wide range for establishing the sperm concentration calibration curve, donor samples were serially diluted at 1‐in‐2, 1‐in‐4, 1‐in‐6, 1‐in‐8, and 1‐in‐10 ratio using a HEPES‐buffered salt solution (117 mM NaCl, 5.3 mM KCl, 1.8 mM CaCl_2_·2H_2_O, 0.8 mM MgSO_4_, 1 mM NaH_2_PO_4_, 5.5 mM D‐glucose, 0.03 mM phenol red, 4 mM NaHCO_3_, 21 mM HEPES, 0.33 mM Na pyruvate, 21.4 mM Na lactate) supplemented with 1 mg/mL polyvinyl alcohol.

### 2.2 Concentration Analysis

**FIGURE 1:**
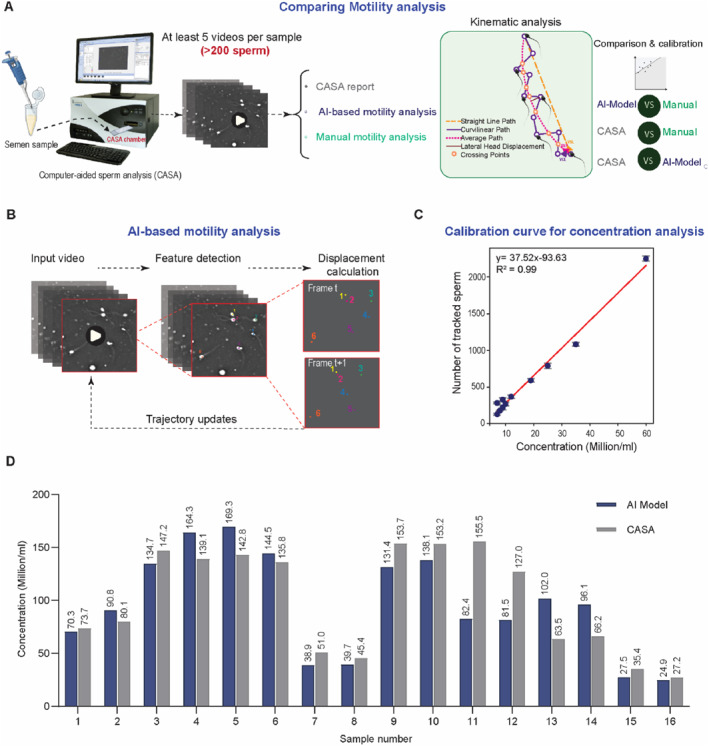
Workflow for sperm motility and concentration analysis. (A) Patient samples were initially assessed for sperm concentration and motility using CASA. The recorded CASA videos were then analyzed using manual tracking in Fiji and our AI‐based tracking model to calibrate and compare calculated motility parameters. (B) The AI‐based tracking model automatically identifies spermatozoa and tracks sperm head centroids in real‐time, assigning a unique ID to each cell for comparing the trajectory between models. (C) Calibration curve for concentration analysis using the number of tracked spermatozoa in the first 10 frames of the video, demonstrating a strong linear correlation (*R* ^2^ = 0.99) with manual measurements. Values are reported as mean ± SD (*n* = 11). (D) Direct comparison of sperm concentration values measured by CASA versus the AI‐based method (*n* = 16).

To establish the calibration curve for sperm concentration (Supporting Information S1: Figure S1), 20 μL of each sample was loaded into a hemocytometer, and spermatozoa were counted manually according to the WHO6 guidelines [19]. In parallel, an aliquot of 5 μL from the same sample was loaded into a MicroCell slide for standardized motility assessment. For each sample, videos were recorded from both chambers of the slide, resulting in two independent recordings per sample. The slides were imaged on an inverted microscope (Olympus IX83, Japan) equipped with an ORCA‐Flash4.0 V3 Digital CMOS camera at 20X objective lens, 50 fps, for 2 s. To enable quantitative comparison with CASA and manual methods, for each sample, both videos were passed through the tracking algorithm, and the number of spermatozoa detected in the first 10 frames of each video was extracted. The average of these two values was calculated and plotted against the corresponding hemocytometer‐based concentration measurement, constructing a concentration calibration curve from model outputs (Figure 1C). Using 10 frames is aligned with the CASA software protocol. This curve was then applied to patient videos analyzed by our tracking model, allowing sperm concentration to be measured and directly compared with CASA‐derived values (Figure 1D).

### 2.3 Sperm Tracking and Motility Analysis

**FIGURE 2:**
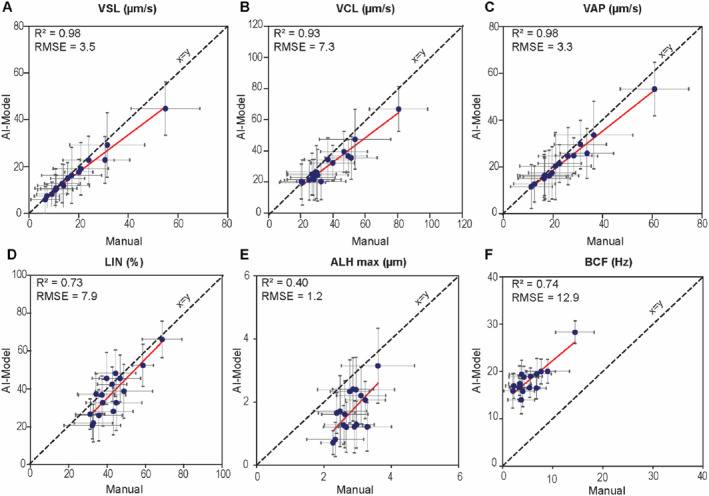
Linear regression analysis comparing motility parameters between the AI‐model and manual tracking. (A) Straight line velocity (VSL), (B) curvilinear velocity (VCL), (C) Average path velocity (VAP), (D) linearity (LIN), (E) maximum amplitude of lateral head displacement (ALH_max_). (F) Beat cross frequency (BCF). Each point represents the average motility parameter per sample (*n* = 16), presented as mean ± SD calculated from 20 spermatozoa per sample in the manual tracking dataset. The solid red line indicates the best‐fit linear regression.

**FIGURE 3:**
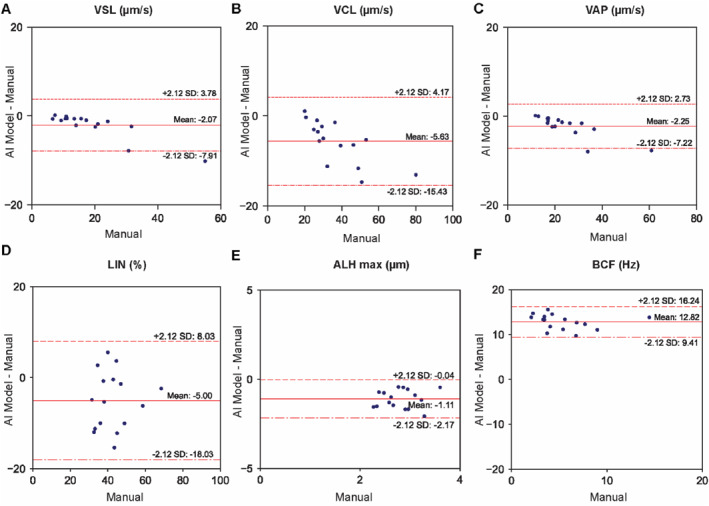
Comparison of population‐level motility parameters between the AI‐model and manual tracking using Bland and Altman plots. (A) VSL, (B) VCL, (C) VAP, (D) LIN, (E) ALH_max_, and (F) BCF (abbreviations as defined for Figure 2). The *x*‐axis shows the values obtained using the reference manual method, while the *y*‐axis shows the difference between the values obtained by the two methods. The solid red line indicates the mean difference, and the dashed red lines represent the mean ± 2.12 SD (for *n* = 16).

We quantified motility in each sample using three methods: (i) a commercial IVOS II CASA system (Software Version 1.16, Hamilton Thorne, Beverly, MA, USA), (ii) our AI‐based tracking algorithm, and (iii) manual tracking in Fiji, which served as ground truth (Figure 1A). In all experiments, well‐mixed patient samples were loaded into a MicroCell slide (Vitrolife Inc., San Diego, CA, USA; 20 μm chamber depth) and processed using the CASA IVOS II system at the Andrology Unit. Specifically, imaging was performed in negative phase‐contrast on the IVOS internal microscope with a Zeiss A‐Plan 10X objective (NA 0.25) and stroboscopic LED illumination at 37°C. Each sample was recorded in two independent drops, with five non‐overlapping fields per drop captured for 100 frames at 60 fps. According to WHO6 [19] guidelines for sperm motility analysis, this approach ensured that >200 spermatozoa were analyzed. For each sample, sperm concentration, CASA‐recorded videos, single‐cell trajectories, and population‐level CASA motility parameters were extracted. To benchmark the model, the extracted videos were analyzed using an adapted optical flow tracking algorithm and compared against laborious manual tracking results, which served as the ground truth to calibrate the motility parameters measured by the model (Figure 2). The results from the calibrated AI model were then compared with those obtained from the CASA (Figure 3).

For AI‐based motility analysis, captured videos were processed with a custom Python script implementing a pyramidal Lucas–Kanade optical‐flow algorithm [47]. The algorithm tracks the centroid of each sperm head, selected as a stable reference point to accurately capture translational motion while minimizing artifacts caused by tail movement. The model includes dynamic detection and reassignment of feature points across frames, with careful optimization of quality thresholds and pyramid levels to ensure trajectory continuity and avoid overlaps between tracked cells. For manual tracking, sperm trajectories were extracted from CASA videos using the manual tracking plugin in the open‐source Fiji software, with 20 spermatozoa tracked per sample. For consistency across tracking methods, each spermatozoon was assigned a unique ID, mapped to its position in the first video frame (Figure 1B, Supporting Information S1: Figure S2).

The resulting trajectory data, whether obtained from AI‐based or manual tracking, were processed using a custom MATLAB script [48] to calculate kinematic metrics typically reported by CASA systems [18]. These include straight‐line velocity (VSL, the distance between the first and last tracked points divided by the time duration); curvilinear velocity (VCL, the sum of the straight line distances between all the points along the track, divided by the corresponding time interval); average path velocity (VAP, the velocity along the smoothed average swimming path); linearity (LIN = VSL/VCL); maximum amplitude of lateral head displacement (ALH_max_, the maximum lateral deviation of the instantaneous 2D trajectory from the averaged path); and beat cross frequency (BCF, the frequency at which the curvilinear trajectory crosses the average path).

### 2.4 Data Analysis

All statistical analyses were performed in Python (v3.9.13) using the SciPy and stats model packages. Manual tracking was used as the benchmark standard to evaluate the accuracy of both the AI‐based computer vision model and the CASA system. For comparison, the same manually tracked spermatozoa were analyzed using the other models, and the resulting motility data were compared (across 16 samples, 320 spermatozoa). Linear regression was then used to calibrate both our concentration analysis and AI‐based motility analysis model. The agreement was quantified using the correlation coefficient (*R* ^2^), RMSE at the sample level, and mean absolute error (MAE) at the single‐cell level. Bland and Altman plots [49, 50] were generated to further assess biases and limits of agreement between methods.

## 3 Results

### 3.1 Concentration Analysis

The AI model accurately estimated sperm concentration from video data using a calibration curve, showing close agreement with CASA measurements.

To establish a method for calculating sperm concentration from video data, we generated a calibration curve by plotting the number of spermatozoa tracked in the first 10 frames by the AI model against manually measured concentrations using a hemocytometer (Figure 1C). A strong linear relationship was observed (*R* ^2^ = 0.99), and the resulting calibration equation was used in our model to calculate sperm concentration from the number of tracked spermatozoa. Figure 1D compares sperm concentrations from the model with CASA measurements for 16 patient samples (not used to establish the calibration curve). Sperm concentrations estimated by the AI model were within 21.9% of CASA measurements, with less than 10% difference for five samples.

### 3.2 Pre‐Calibration Performance

The AI model showed strong agreement with manual tracking for key motility parameters (VSL, VCL, VAP), outperforming CASA, while moderate agreement was observed for the derived kinematic values BCF and ALH_max_.

Sperm motility parameters obtained from the AI‐based tracking model were compared against both manual tracking (used as the ground truth) and the CASA system at both the single‐cell and the population levels (i.e., average motility values per sample). Figure 2 presents a comparative analysis between the AI model and manual tracking at the population level. The AI model demonstrated strong linear correlations with manual tracking for key velocity parameters, including VSL (*R* ^2^ = 0.98, RMSE = 3.5 μm/s), VCL (*R* ^2^ = 0.93, RMSE = 7.3 μm/s), and VAP (*R* ^2^ = 0.98, RMSE = 3.3 μm/s). Moderate and weaker correlations were observed for LIN (*R* ^2^ = 0.73, RMSE = 7.9%), ALH_max_ (*R* ^2^ = 0.40, RMSE = 1.2 μm), and BCF (*R* ^2^ = 0.74, RMSE = 12.9 Hz), suggesting that ALH and BCF may be more sensitive to variations in tracking resolution and methodology, including how the average path is computed, which can change crossing counts with the instantaneous trajectory (Supporting Information S1: Figure S5). Bland and Altman plots (Figure 3) revealed narrow limits of agreement for VSL, VCL, VAP, and LIN, with mean differences close to zero, indicating strong consistency between the model and manual tracking. However, for ALH_max_ and BCF, the AI model exhibited larger mean differences (−1.11 μm and 12.82 Hz, respectively) and wider limits of agreement (−2.17 to −0.04 μm for ALH_max_ and 9.41–16.24 Hz for BCF), with ALH_max_ slightly underestimated and BCF substantially overestimated by the AI model. At the single‐cell level (Supporting Information S1: Figures S3 and S4), a similar trend was observed, but with some reduction in correlation strength for VSL (*R* ^2^ = 0.89, RMSE = 7.4 μm/s), VCL (*R* ^2^ = 0.83, RMSE = 14.9 μm/s), VAP (*R* ^2^ = 0.87, RMSE = 8.9 μm/s), and LIN (*R* ^2^ = 0.65, RMSE = 14.6%). At the single‐cell level, ALH_max_ showed a slightly improved correlation (*R* ^2^ = 0.45, RMSE = 1.5 μm), whereas BCF remained highly variable with low correlation and wide dispersion. To understand the discrepancy in BCF estimates, we examined sperm trajectories exhibiting the largest differences between model and manual tracking (Supporting Information S1: Figures S5 and S6). These inconsistencies were particularly evident in spermatozoa exhibiting tight circular motion (with radii of only a few microns) or very low motility. In these cases, the instantaneous trajectory and the resulting smoothed average path diverge significantly between manual tracking and the automated methods, leading to a systematic overestimation of BCF by both the CASA system and the AI model.

Similar analysis was conducted for the CASA system, with comparisons to manual tracking at both the population level (Supporting Information S1: Figures S7 and S8) and single‐cell level (Supporting Information S1: Figures S9 and S10). CASA exhibited comparable trends but generally showed lower agreement with the manual data than the AI model for key velocity metrics, including VSL, VCL, and VAP. Notably, BCF from CASA was significantly overestimated, with weak correlation to manual tracking at the population level (*R* ^2^ = 0.41, RMSE = 17.7 Hz, Supporting Information S1: Figure S7) and no observable correlation at the single‐cell level (*R* ^2^ = 0.00, RMSE = 31.7 Hz, Supporting Information S1: Figure S9). CASA frequently reported implausibly high BCF values, approaching 100 Hz, even for nearly immotile cells, highlighting a key limitation of its tracking algorithm (Supporting Information S1: Figure S9). Interestingly, BCF values reported by CASA and the AI model showed closer alignment with each other (Supporting Information S1: Figure S6), further suggesting that discrepancies with manual tracking arise from fundamental limitations in how these automated systems interpret motion in low‐motility or circular‐swimming spermatozoa. To address this limitation, we implemented a thresholding rule in the AI model that for cells with VSL ≤ 2 μm/s, BCF was not reported, based on the rationale that such low velocities yield minimal distinction between instantaneous and average swimming paths, rendering BCF estimation unreliable in these cases.

### 3.3 Post‐Calibration Performance

The calibrated AI model showed strong consistency with manual tracking for most motility parameters (VSL, VCL, VAP, LIN, ALH_max_), with improvements in the accuracy of ALH_max_ and BCF.

To improve the accuracy of our AI model, we applied calibration equations derived from single‐cell comparisons between model outputs and manual tracking (Supporting Information S1: Figure S3). After calibration, performance at the single‐cell level (Supporting Information S1: Figures S11 and S12) remained comparable to the original model for most motility parameters, with similar regression coefficients and RMSE values. At the population level, calibrated outputs for VSL, VCL, VAP, and LIN also closely matched those from the uncalibrated model, but with slight improvements in RMSE (Supporting Information S1: Figure S13) and mean differences that were closer to zero accompanied by narrower limits of agreement (Supporting Information S1: Figure S14). Most notably, calibration yielded substantial improvements in the evaluation of ALH_max_ and BCF. Specifically, the RMSE for ALH_max_ improved by 30% (from 1.2 to 0.84 μm) and for BCF by 52% (from 12.9 to 6.17 Hz). The mean difference between manual and calibrated AI model for these parameters also decreased markedly, by 46% for ALH_max_ (from −1.11 to −0.60 μm) and 66% for BCF (from 12.82 to 4.31 Hz), alongside much narrower limits of agreement, indicating increased reliability (Supporting Information S1: Figure S14).

**FIGURE 4:**
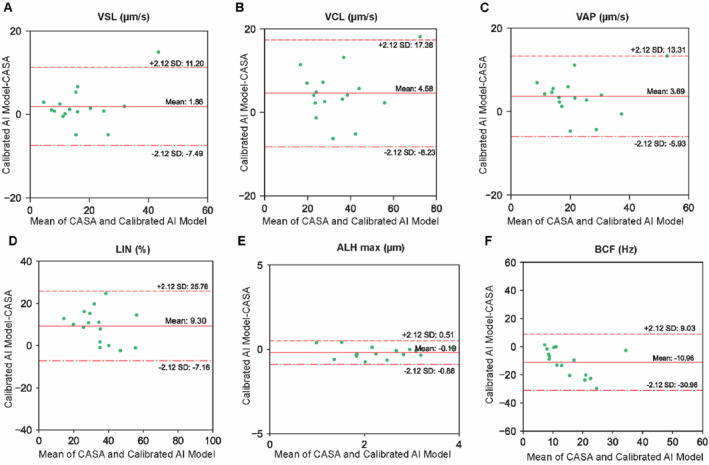
Comparison of population‐level motility parameters between the calibrated AI model and CASA using Bland and Altman plots. (A) VSL, (B) VCL, (C) VAP, (D) LIN, (E) ALH_max_, and (F) BCF (abbreviations as defined for Figure 2). The *x*‐axis represents the mean value from the two methods, while the *y*‐axis shows the difference between values from the two methods. The solid red line indicates the mean difference, and the dashed red lines represent the 95% range (i.e., mean ± 2.12 SD).

When comparing the calibrated AI model to CASA (Figure 3), strong correlations were observed for key motility parameters, including VSL (*R* ^2^ = 0.85, RMSE = 4.8 μm/s), VCL (*R* ^2^ = 0.84, RMSE = 6.2 μm/s), VAP (*R* ^2^ = 0.84, RMSE = 5.8 μm/s), and ALH_max_ (*R* ^2^ = 0.82, RMSE = 0.4 μm). A weaker correlation was observed for LIN (*R* ^2^ = 0.64, RMSE = 12.1%). However, as above, BCF values reported by CASA were considerably scattered and overestimated relative to the calibrated AI model, with a large mean difference of −10.96 Hz and wide limits of agreement (Figure 4). Comparisons at the single‐cell level (Supporting Information S1: Figures S15 and S16) revealed similar trends; after calibration, correlations with CASA decreased, especially for BCF, reflecting the model's correction of CASA instabilities rather than replicating them. Collectively, these results indicate that CASA performs poorly in deriving BCF for some trajectories, a limitation that is substantially corrected by the calibration of our AI model, resulting in BCF and ALH_max_ values that are in much closer agreement with manual tracking. Overall, the calibrated AI model provided a reliable and robust alternative to CASA for sperm kinematics.

### 3.4 Motility Grading Analysis

The calibrated AI model showed strong agreement with manual tracking and more accurate grading of sperm motility categories than CASA, closely matching manual assessments across rapid, slow, and non‐progressive groups.

The clinical grading of sperm motility is essential for predicting male fertility potential and guiding treatment decisions, as it directly reflects the ability of spermatozoon to reach and fertilize an oocyte [51]. According to WHO6 [19], sperm motility is categorized into four distinct grades based on VSL: rapid progressive (VSL ≥ 25 µm/s), slow progressive (5 μm/s ≤ VSL < 25 μm/s), non‐progressive (VSL < 5 μm/s), and immotile (no active movement). Of these categories, the proportion of rapid progressive spermatozoa is particularly critical, as it serves as a direct indicator of a sample's capacity for natural fertilization following successful navigation through the female reproductive tract [52]. Higher percentages of rapidly progressive spermatozoa are consistently associated with improved fertility outcomes [52]. This is especially important for penetrating cervical mucus, the essential first step for natural conception [53]. Progressive motility also determines success in assisted reproduction; the percentage of progressively motile spermatozoa yields better fertilization outcomes in conventional IVF [8]. Many clinics use progressive motility thresholds to determine whether conventional IVF is likely to succeed. If motility falls below this threshold, ICSI is often selected to bypass the limited sperm fertilizing capacity [54].

**FIGURE 5:**
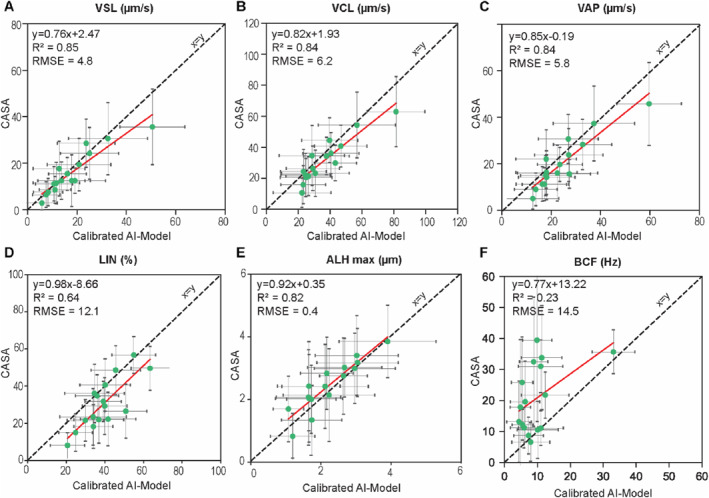
Comparing motility parameters between the calibrated AI‐model and CASA. (A) VSL, (B) VCL, (C) VAP, (D) LIN, (E) ALH_max_, and (F) BCF (abbreviations as defined for Figure 2). Each point represents the average motility parameter per sample (*n* = 16), presented as mean ± SD calculated from 20 spermatozoa per sample. The solid red line indicates the best‐fit linear regression.

**FIGURE 6:**
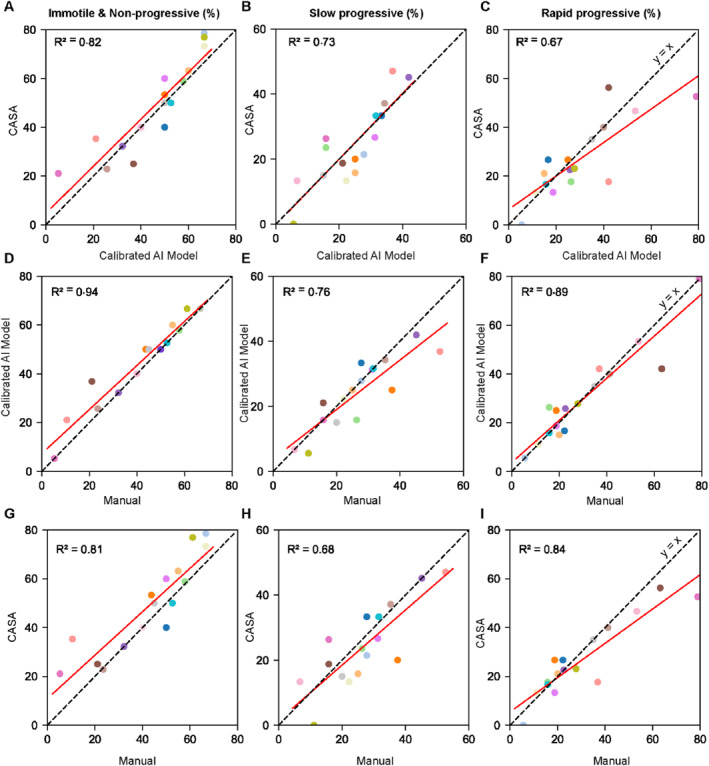
Comparison of sperm motility grading across methods. The percentage of (A) immotile and non‐progressive, (B) slow progressive, and (C) rapid progressive sperm for CASA versus the calibrated AI model, (D–F) for the calibrated AI model versus manual tracking, and (G–I) for CASA versus manual tracking. Each color represents data from an individual semen sample. The solid red line indicates the regression fit.

Figure 5 presents a comparison of sperm motility grading between the calibrated AI model and CASA for 16 patient samples, with full comparative data, including manual tracking, provided in Supporting Information S1: Table S1 and Figure 6. The results are reported as the percentages of rapid progressive, slow progressive, and the combined group of immotile and non‐progressive (IM&NP) spermatozoa for each sample. While both the calibrated AI model and CASA demonstrated substantial correlation in motility grading across these categories (*R* ^2^ ranging from 0.67 to 0.82; Figure 5), the calibrated AI model consistently outperformed CASA when benchmarked against manual tracking (Figure 6). Specifically, the AI model showed strong linear correlation with manual tracking for the percentage of rapid progressive (*R* ^2^ = 0.89), slow progressive (*R* ^2^ = 0.76), and IM&NP (*R* ^2^ = 0.94) spermatozoa. In contrast, CASA exhibited more scattered results, with lower *R* ^2^ values of 0.84, 0.68, and 0.81 for these respective categories.

Analysis of grading accuracy (Supporting Information S1: Table S1) further highlighted these differences. The calibrated AI model on average underestimated the percentage of NP&IM sperm by 14% (vs. −41% for CASA), overestimated slow progressive spermatozoa by 8% (vs. 7% for CASA), and underestimated rapid progressive spermatozoa by only 3% (whereas CASA overestimated by 8%). Notably, for the 16 samples tested, the calibrated AI model provided motility grading results identical to manual tracking in 6 samples, compared to only 1 sample for CASA. These findings collectively highlight the reliable and accurate performance of the calibrated AI model for sperm motility grading. Importantly, the tendency of CASA to underestimate non‐progressive and immotile spermatozoa and overestimate rapid progressive spermatozoa could lead to misclassification of semen quality, potentially affecting patient classification and treatment decisions. By providing results that closely align with manual tracking, the calibrated AI model would support more accurate clinical assessment to improve fertility care.

### 3.5 Repeatability Analysis

The calibrated AI model demonstrated higher repeatability and robustness than CASA, showing smaller variations across replicate samples and consistent performance under changes in video brightness and orientation.

**FIGURE 7:**
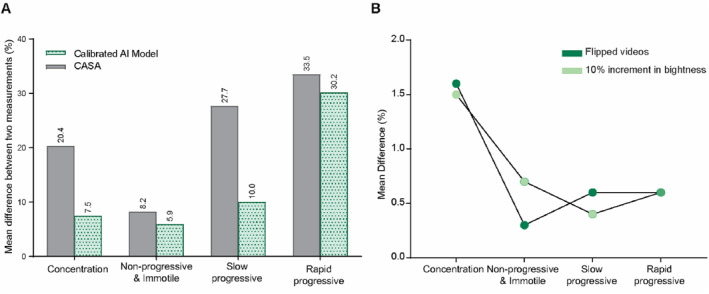
Repeatability and robustness of the calibrated AI model. (A) The mean percentage difference between repeated measurements for each sample, calculated as the average of mean difference values reported in Table 1 across six tested samples, is presented for both the calibrated AI model and CASA. (B) The impact of video alterations, including a 10% increase in brightness and image flipping, on the performance of the calibrated AI model.

To assess repeatability, we compared the performance of the calibrated AI model and CASA in measuring sperm concentration and motility grading across six new patient samples, each analyzed in duplicate (Table 1). Although both methods produced comparable results, the calibrated AI model demonstrated superior repeatability, as reflected by lower mean differences between repeated measurements. Specifically, the calibrated AI model outperformed CASA by achieving, on average, 12.9% (7.5% vs. 20.4%), 2.3% (5.9% vs. 8.2%), 17.7% (10.0% vs. 27.7%), and 3.3% (30.2% vs. 33.5%) smaller mean differences in assessing sperm concentration, and the percentages of IM&NP, slow progressive, and rapid progressive spermatozoa, respectively (Figure 7A). These findings indicate that the calibrated AI model yields more consistent results than CASA when analyzing replicate samples.

**TABLE 1:** Repeatability of the calibrated AI model compared with CASA.

Sample number	Measurement	Concentration (M/mL)				Non‐progressive and immotile (%)				Slow progressive (%)				Rapid progressive (%)			
		CASA		Model		CASA		Model		CASA		Model		CASA		Model	
1	1	156.6	**6.3%**	151.5	1.6%	41.4	**27.1%**	39.6	20.6%	6.9	**52.4%**	19.6	10.6%	51.7	**41.9%**	40.8	32.1%
	2	147.1		149.1		54.4		48.7		11.8		21.8		33.8		29.5	
2	1	47.3	0.4%	41.2	**11.9%**	79.9	**7.4%**	74.7	**10.7%**	6.5	15.6%	14.7	15.6%	13.5	30.2%	10.6	**38.8%**
	2	47.1		46.4		74.2		67.1		7.6		17.2		18.3		15.7	
3	1	13.2	**53.7%**	29.4	8.8%	70.6	**10.0%**	84.8	0.9%	12.2	**19.8%**	11.3	9.3%	17.1	**35.9%**	3.9	8.0%
	2	22.9		32.1		78.0		84.0		10		12.4		11.9		3.6	
4	1	40.4	3.6%	38.4	**8.4%**	93.3	0.7%	93.4	**1.0%**	4.4	**41.1%**	6.1	7.9%	2.3	32.7%	0.5	**66.7%**
	2	41.9		41.8		94		92.4		2.9		6.6		3.2		1.0	
5	1	203.7	**50.1%**	216.7	10.9%	81.8	**4.1%**	80.3	1.6%	6.4	**8.1%**	15.4	3.2%	11.8	**28.0%**	4.3	20.5%
	2	339.8		241.8		85.2		81.6		5.9		14.9		8.9		3.5	
6	1	9.1	**8.0%**	8.7	3.5%	91.8	0.0%	92.4	**1.1%**	5.1	**29.2%**	4.7	13.6%	3.1	**32.4%**	2.9	14.8%
	2	8.4		8.4		91.8		93.4		3.8		4.1		4.3		2.5	

*Note:* Sperm concentration and motility grading results were obtained for six new patient samples, each analyzed in duplicate by both the calibrated AI model and CASA. For each sample, vertical values in the percentage difference between the two repeated measurements for the corresponding method, calculated as Percentage difference = 100 × ∣Measurement_1_ − Measurement_2_∣/Average of Measurement_1_ and Measurement_2_. Bold values indicate the higher percentage difference between duplicate measurements for the same sample, indicating whether CASA or the Model shows greater variability.

We further evaluated the robustness of the calibrated AI model to changes in video acquisition conditions by introducing a 10% increase in brightness and applying image flipping (Figure 7B). Across all concentration and motility grading parameters, mean differences remained below 2%, indicating that the model's performance was minimally affected by such alterations. More specifically, the mean difference for concentration analysis was ∼1.5%, and less than 0.75% for all motility grading categories. Additionally, the model allows for adjustable frame rates and video lengths. These results confirm the high repeatability and robustness of the calibrated AI model.

## 4 Discussion

Accurate assessment of sperm motility and concentration remains fundamental to the diagnosis and management of male infertility, directly influencing both clinical decision‐making and treatment success in reproductive medicine [5, 6, 12, 15, 19]. Recent AI studies either performed sample‐level motility prediction, using supervised or unsupervised tracking and feature quantization, benchmarked to visual grading [39, 43], or paired deep object detection with modified trackers, emphasizing high detection and tracking on short sequences [37, 55] (Supporting Information S1: Table S2). In this study, we introduce an AI‐driven computer vision tool that provides robust, quantitative, and reproducible analysis of sperm motility and concentration, and is rigorously benchmarked against manual tracking and compared with commercial CASA systems. Our findings demonstrate that the calibrated AI model outperforms CASA in terms of measurement reliability. Manual sperm tracking has long been considered the gold standard for precise motility analysis [56], but it is not feasible for routine clinical practice due to its labor‐intensive and time‐consuming nature. Our AI‐based tracking model addresses these limitations by showing strong linear correlation with manual tracking for key motility parameters (sperm kinematics), including VSL, VCL, and VAP (population‐level *R* ^2^ ≥ 0.93; RMSE: 3.3–7.3 μm/s). Lower agreement for ALH_max_ and BCF reflects the sensitivity of these motility parameters to tracking length and frame rate [57], the dependence of BCF on how the average path is estimated [19], and the sub‐micron head motion that manual pixel‐level tracking cannot resolve (Supporting Information S1: Figure S6). These correlations suggest a high accuracy of our model in capturing the kinematic features essential for male fertility assessment.

Importantly, our results suggest that CASA, despite its widespread adoption and advantages in standardization and throughput, suffers from systematic errors, especially in the estimation of BCF and motility grading. CASA frequently overestimated BCF, even in immotile spermatozoa, and exhibited higher RMSE (5.5–14.8) values for key motility metrics compared to our AI model. This highlights the key advantage of our AI model, which could enable more accurate assessment of motility parameters, including BCF, for the precise identification and classification of clinically relevant sperm subpopulations, thereby potentially supporting more informed and effective clinical decision‐making. A significant strength of our study is the calibration of the AI model against manual tracking. Calibration particularly improved the assessment of ALH_max_ and BCF at the population level, metrics known to be sensitive to tracking methodology and imaging artifacts. For instance, RMSE for ALH_max_ improved by 30%, and for BCF by 52% post‐calibration, with the mean differences and limits of agreement narrowing substantially. Nonetheless, our findings also highlight the inherent limitations of BCF as a motility parameter, particularly under standard 60 fps acquisition, where aliasing [58] may contribute to its overestimation, an important consideration when interpreting frequency‐based metrics. This approach not only ensures that our model aligns closely with manual tracking results but also enhances clinical trustworthiness and reproducibility, two features often lacking in earlier AI‐based or automated systems.

The calibrated AI model also demonstrates potential for application in sperm motility grading, an important step in assessing fertility potential and informing treatment decisions [8, 19, 54]. The model demonstrated a strong correlation with manual grading across all motility categories (*R* ^2^ up to 0.94 for IM&NP sperm and 0.89 for rapid progressive sperm), consistently outperforming CASA, which showed greater scatter and lower correlation coefficients in direct comparison. Notably, our model avoided the tendency of CASA to underestimate non‐progressive and immotile spermatozoa and to overestimate rapid progressive fractions, trends that risk misclassifying semen quality and could adversely affect patient management.

Repeatability and robustness are essential for clinical implementation. Our results showed that the AI model also had substantially lower mean differences between repeated measurements of both sperm concentration and motility grading categories on the same sample compared with CASA. The mean difference for sperm concentration, for example, was reduced from 20.4% to 7.5%, a decrease of 12.9%; and for slow progressive motility, from 27.7% to 10%, a decrease of 17.7%. Notably, the greatest discrepancies between repeated CASA measurements were observed in high‐concentration samples, where CASA‐reported values could differ by more than 130 M/mL (Sample 5). In contrast, the AI model demonstrated significantly greater consistency, with a corresponding difference of ∼25 M/mL for the same sample. This instability in CASA measurements at higher concentrations may be attributed to known limitations outlined in the CASA system manual, which states that accuracy decreases significantly when sperm concentration exceeds 30 M/mL. The model's performance remained stable under variations in imaging conditions, including adjustments in brightness and image orientation, with mean differences in output consistently below 2%. Collectively, this demonstrates the repeatability and robustness of the AI model, supporting its potential use across laboratories with varying equipment and operator experience.

In summary, we present a robust, accurate, and clinically adaptable AI‐based tool for sperm motility and concentration analysis. Our approach overcomes key limitations of CASA and manual tracking methods, providing highly reproducible and quantitative results. We provide comprehensive kinematic and motility analysis at both the single‐cell and population levels, demonstrating strong reproducibility. In repeat tests and varying imaging conditions, the calibrated model maintained stable performance and showed superior repeatability to CASA, and given its compatibility with multiple imaging modes and acquisition settings, it has clear potential for clinical implementation. To avoid debris tracking, we restricted features to high‐contrast corners, and also followed standard CASA sample acquisition rules and excluded fields with bubbles and aggregates. While our model was evaluated against both manual tracking and CASA, further multi‐center validation on larger, demographically diverse cohorts will be needed for full validation. Our current AI model performs optimally on videos acquired with negative phase‐contrast or DIC imaging at 10× or 20× magnification and 50–60 fps. Evaluation of generalizability across different imaging systems and acquisition settings (e.g., frame rate, magnification, illumination) will also be necessary.

## Author Contributions

S.S. and R.N. designed the study. G.A. provided clinical supervision and contributed to data collection. G.A., D.M., M.K.O.B., R.M., and D.Z.F. designed and supervised the clinical aspects of the work. K.A. and A.N. provided supervision for the computational modeling and analysis of the data. S.S. performed the research and developed the model. All authors reviewed the data and contributed to the preparation and revision of the manuscript.

## Ethics Statement

This study was approved by the Monash University Human Research Ethics Committee.

## Consent

All participants provided written informed consent before sample collection. No animal experiments were conducted in this study. Patient privacy was protected by assigning randomly generated de‐identified numbers to the samples.

## Conflicts of Interest

The authors declare no conflicts of interest.

## Supporting information


Supporting Information S1

## Data Availability

The datasets and video materials supporting the findings of this study are not publicly available but may be obtained from the corresponding author, Reza Nosrati (reza.nosrati@monash.edu), upon reasonable request. The analysis code and models were developed in Python (version 3.9.13) but are not publicly available due to potential commercial interests and intellectual property considerations.

## References

[1] A. Agarwal , A. Mulgund , A. Hamada , and M. R. Chyatte , “A Unique View on Male Infertility Around the Globe,” Reproductive Biology and Endocrinology 13 (2015): 37. 25928197 10.1186/s12958-015-0032-1 PMC4424520

[2] M. L. Eisenberg , S. C. Esteves , D. J. Lamb , et al., “Male Infertility,” Nature Reviews Disease Primers 9 (2023): 49. 10.1038/s41572-023-00459-w 37709866

[3] C. J. De Jonge , C. L. R. Barratt , R. J. Aitken , et al., “Current Global Status of Male Reproductive Health,” Human Reproduction Open 2024 (2024): hoae017. 38699533 10.1093/hropen/hoae017 PMC11065475

[4] Y. Zhu , B. Kong , R. Liu , and Y. Zhao , “Developing Biomedical Engineering Technologies for Reproductive Medicine,” Smart Medicine 1 (2022): e20220006. 39188735 10.1002/SMMD.20220006 PMC11235786

[5] E. T. Donnelly , S. E. M. Lewis , J. A. McNally , and W. Thompson , “In Vitro Fertilization and Pregnancy Rates: The Influence of Sperm Motility and Morphology on IVF Outcome,” Fertility and Sterility 70 (1998): 305. 9696226 10.1016/s0015-0282(98)00146-0

[6] G. M. Buck Louis , R. Sundaram , E. F. Schisterman , et al., “Semen Quality and Time to Pregnancy: The Longitudinal Investigation of Fertility and the Environment Study,” Fertility and Sterility 101 (2014): 453. 24239161 10.1016/j.fertnstert.2013.10.022 PMC3946620

[7] P. Fernández‐López , J. Garriga , I. Casas , M. Yeste , and F. Bartumeus , “Predicting Fertility From Sperm Motility Landscapes,” Communications Biology 5 (2022): 1027. 36171267 10.1038/s42003-022-03954-0 PMC9519750

[8] M. T. Villani , D. Morini , G. Spaggiari , et al., “Are Sperm Parameters Able to Predict the Success of Assisted Reproductive Technology? A Retrospective Analysis of Over 22,000 Assisted Reproductive Technology Cycles,” Andrology 10 (2022): 310. 34723422 10.1111/andr.13123 PMC9298690

[9] D. Ren , B. Navarro , G. Perez , et al., “A Sperm Ion Channel Required for Sperm Motility and Male Fertility,” Nature 413 (2001): 603. 11595941 10.1038/35098027 PMC8462998

[10] H. Wang , L. L. McGoldrick , and J. J. Chung , “Sperm Ion Channels and Transporters in Male Fertility and Infertility,” Nature Reviews Urology 18 (2021): 46. 33214707 10.1038/s41585-020-00390-9 PMC7852504

[11] K. Kerns , M. Zigo , E. Z. Drobnis , M. Sutovsky , and P. Sutovsky , “Zinc Ion Flux During Mammalian Sperm Capacitation,” Nature Communications 9 (2018): 2061. 10.1038/s41467-018-04523-y PMC5970269 29802294

[12] V. Q. Dang , L. N. Vuong , T. M. Ho , et al., “The Effectiveness of ICSI Versus Conventional IVF in Couples With Non‐Male Factor Infertility: Study Protocol for a Randomised Controlled Trial,” Human Reproduction Open 2019 (2019): hoz006. 30937394 10.1093/hropen/hoz006 PMC6436611

[13] S. Bhattacharya , M. Hamilton , M. Shaaban , et al., “Conventional In‐Vitro Fertilisation Versus Intracytoplasmic Sperm Injection for the Treatment of Non‐Male‐Factor Infertility: A Randomised Controlled Trial,” Lancet 357 (2001): 2075. 11445099 10.1016/s0140-6736(00)05179-5

[14] J. Zheng , Y. Lu , X. Qu , et al., “Decreased Sperm Motility Retarded ICSI Fertilization Rate in Severe Oligozoospermia but Good‐Quality Embryo Transfer Had Achieved the Prospective Clinical Outcomes,” PLoS One 11 (2016): e0163524. 27661081 10.1371/journal.pone.0163524 PMC5035010

[15] A. Muthigi , S. Jahandideh , L. A. Bishop , et al., “Clarifying the Relationship Between Total Motile Sperm Counts and Intrauterine Insemination Pregnancy Rates,” Fertility and Sterility 115 (2021): 1454. 33610321 10.1016/j.fertnstert.2021.01.014

[16] P. Vogiatzi , A. Pouliakis , M. Sakellariou , et al., “Male Age and Progressive Sperm Motility Are Critical Factors Affecting Embryological and Clinical Outcomes in Oocyte Donor ICSI Cycles,” Reproductive Sciences 29 (2022): 883. 34782988 10.1007/s43032-021-00801-1

[17] C. Gnoth , B. Maxrath , T. Skonieczny , K. Friol , E. Godehardt , and J. Tigges , “Final ART Success Rates: A 10 Years Survey,” Human Reproduction 26 (2011): 2239. 21659314 10.1093/humrep/der178

[18] D. Mortimer , L. Björndahl , C. L. R. Barratt , et al., A Practical Guide to Basic Laboratory Andrology, 2nd ed. (Cambridge University Press, 2022).

[19] World Health Organization , WHO Laboratory Manual for the Examination and Processing of Human Semen, 6th ed. (World Health Organization, 2021).

[20] J. Auger , F. Eustache , B. Ducot , et al., “Intra‐ and Inter‐Individual Variability in Human Sperm Concentration, Motility and Vitality Assessment During a Workshop Involving Ten Laboratories,” Human Reproduction 15 (2000): 2360. 11056133 10.1093/humrep/15.11.2360

[21] S. Sikka and W. Hellstrom , “Current Updates on Laboratory Techniques for the Diagnosis of Male Reproductive Failure,” Asian Journal of Andrology 18 (2016): 392–401. 27056346 10.4103/1008-682X.179161 PMC4854088

[22] R. Finelli , K. Leisegang , S. Tumallapalli , R. Henkel , and A. Agarwal , “The Validity and Reliability of Computer‐Aided Semen Analyzers in Performing Semen Analysis: A Systematic Review,” Translational Andrology and Urology 10 (2021): 3069. 34430409 10.21037/tau-21-276 PMC8350227

[23] L. F. C. Brito , G. C. Althouse , C. Aurich , et al., “Andrology Laboratory Review: Evaluation of Sperm Concentration,” Theriogenology 85 (2016): 1507. 27045626 10.1016/j.theriogenology.2016.01.002

[24] L. Björndahl , C. L. R. Barratt , D. Mortimer , et al., “Standards in Semen Examination: Publishing Reproducible and Reliable Data Based on High‐Quality Methodology,” Human Reproduction 37 (2022): 2497. 36112046 10.1093/humrep/deac189 PMC9627864

[25] J. Lammers , S. Chtourou , A. Reignier , S. Loubersac , P. Barrière , and T. Fréour , “Comparison of Two Automated Sperm Analyzers Using 2 Different Detection Methods Versus Manual Semen Assessment,” Journal of Gynecology Obstetrics and Human Reproduction 50 (2021): 102084. 33545411 10.1016/j.jogoh.2021.102084

[26] W. E. Maalouf and M. Tomlinson , “Computer Assisted Sperm Analysis (CASA),” in Mastering Clinical Embryology: Good Practice, Clinical Biology, Assisted Reproductive Technologies, and Advanced Laboratory Skills, ed. A. Campbell and W. Maalouf (CRC Press, 2024), 244–249.

[27] M. J. Tomlinson , K. Pooley , T. Simpson , et al., “Validation of a Novel Computer‐Assisted Sperm Analysis (CASA) System Using Multitarget‐Tracking Algorithms,” Fertility and Sterility 93 (2010): 1911. 19200972 10.1016/j.fertnstert.2008.12.064

[28] S. T. Mortimer , G. van der Horst , and D. Mortimer , “The Future of Computer‐Aided Sperm Analysis,” Asian Journal of Andrology 17 (2015): 545. 25926614 10.4103/1008-682X.154312 PMC4492043

[29] L. Hackerova , A. Pilsova , Z. Pilsova , et al., “Boar Sperm Motility Assessment Using Computer‐Assisted Sperm Analysis: Current Practices, Limitations, and Methodological Challenges,” Animals 15 (2025): 305. 39943075 10.3390/ani15030305 PMC11816302

[30] D. Mortimer and S. T. Mortimer , “Routine Application of CASA in Human Clinical Andrology and ART Laboratories,” in XIIIth International Symposium on Spermatology, ed. L. Björndahl , J. Flanagan , R. Holmberg , and U. Kvist (Springer, 2021), 183–197.

[31] E. Moen , D. Bannon , T. Kudo , W. Graf , M. Covert , and D. Van Valen , “Deep Learning for Cellular Image Analysis,” Nature Methods 16 (2019): 1233. 31133758 10.1038/s41592-019-0403-1 PMC8759575

[32] B. Chen , Z. Yin , B. W. L. Ng , et al., “Label‐Free Live Cell Recognition and Tracking for Biological Discoveries and Translational Applications,” npj Imaging 2 (2024): 41. 40603709 10.1038/s44303-024-00046-y PMC12118707

[33] J. B. You , C. McCallum , Y. Wang , J. Riordon , R. Nosrati , and D. Sinton , “Machine Learning for Sperm Selection,” Nature Reviews Urology 18 (2021): 387. 34002070 10.1038/s41585-021-00465-1

[34] S. Shahali , F. Akbaridoust , A. Neild , and R. Nosrati , “Advancements in Microfluidic Technologies for Male Infertility,” Advanced Materials Technologies 10 (2025): 2401520.

[35] C. Dai , Z. Zhang , G. Shan , et al., “Advances in Sperm Analysis: Techniques, Discoveries and Applications,” Nature Reviews Urology 18 (2021): 447. 34075227 10.1038/s41585-021-00472-2

[36] L. F. Urbano , P. Masson , M. VerMilyea , and M. Kam , “Automatic Tracking and Motility Analysis of Human Sperm in Time‐Lapse Images,” IEEE Transactions on Medical Imaging 36 (2017): 792. 27875219 10.1109/TMI.2016.2630720

[37] M. R. Mohammadi , M. Rahimzadeh , and A. Attar , “Sperm Detection and Tracking in Phase‐Contrast Microscopy Image Sequences Using Deep Learning and Modified CSR‐DCF,” preprint, arXiv, April 4, 2020, 10.48550/arXiv.2002.04034.

[38] P. Hidayatullah , T. L. E. R. Mengko , R. Munir , and A. Barlian , “Bull Sperm Tracking and Machine Learning‐Based Motility Classification,” IEEE Access 9 (2021): 61159.

[39] S. Ottl , S. Amiriparian , M. Gerczuk , and B. W. Schuller , “motilitAI: A Machine Learning Framework for Automatic Prediction of Human Sperm Motility,” iScience 25 (2022): 104644. 35856034 10.1016/j.isci.2022.104644 PMC9287611

[40] M. Hürland , D. A. Kuhlgatz , C. Kuhlgatz , J. H. Osmers , M. Jung , and M. Schulze , “The Use of Machine Learning Methods to Predict Sperm Quality in Holstein Bulls,” Theriogenology 197 (2023): 16. 36462332 10.1016/j.theriogenology.2022.11.032

[41] K. A. Hook , Q. Yang , L. Campanello , W. Losert , and H. S. Fisher , “The Social Shape of Sperm: Using an Integrative Machine‐Learning Approach to Examine Sperm Ultrastructure and Collective Motility,” Proceedings of the Royal Society B 288 (2021): 20211553. 34547913 10.1098/rspb.2021.1553 PMC8456146

[42] A. Mehrjerd , T. Dehghani , M. Jajroudi , S. Eslami , H. Rezaei , and N. K. Ghaebi , “Ensemble Machine Learning Models for Sperm Quality Evaluation Concerning Success Rate of Clinical Pregnancy in Assisted Reproductive Techniques,” Scientific Reports 14 (2024): 24283. 39414869 10.1038/s41598-024-73326-7 PMC11484743

[43] T. B. Haugen , O. Witczak , S. A. Hicks , L. Björndahl , J. M. Andersen , and M. A. Riegler , “Sperm Motility Assessed by Deep Convolutional Neural Networks Into WHO Categories,” Scientific Reports 13 (2023): 14777. 37679484 10.1038/s41598-023-41871-2 PMC10484948

[44] D. Somasundaram and M. Nirmala , “Faster Region Convolutional Neural Network and Semen Tracking Algorithm for Sperm Analysis,” Computer Methods and Programs in Biomedicine 200 (2021): 105918. 33465511 10.1016/j.cmpb.2020.105918

[45] R. Zhu , Y. Cui , E. Hou , and J. Huang , “Efficient Detection and Robust Tracking of Spermatozoa in Microscopic Video,” IET Image Processing 15 (2021): 3200.

[46] L. Noy , I. Barnea , M. Dudaie , D. Kamber , M. Levi , and N. T. Shaked , “Location Prediction of Sperm Cells Using Long Short‐Term Memory Networks,” Advanced Intelligent Systems 5 (2023): 2300161.

[47] H. Yedjour , “Optical Flow Based on Lucas‐Kanade Method for Motion Estimation,” in Artificial Intelligence and Renewables Towards an Energy Transition, ed. M. Hatti (Springer, 2021), 937–945.

[48] R. Nosrati , A. Driouchi , C. M. Yip , and D. Sinton , “Two‐Dimensional Slither Swimming of Sperm Within a Micrometre of a Surface,” Nature Communications 6 (2015): 8703. 10.1038/ncomms9703 PMC4667638 26555792

[49] J. M. Bland and D. G. Altman , “Statistical Methods for Assessing Agreement Between Two Methods of Clinical Measurement,” International Journal of Nursing Studies 47 (2010): 931.

[50] M. A. Mansournia , R. Waters , M. Nazemipour , M. Bland , and D. G. Altman , “Bland‐Altman Methods for Comparing Methods of Measurement and Response to Criticisms,” Global Epidemiology 3 (2021): 100045. 37635723 10.1016/j.gloepi.2020.100045 PMC10446118

[51] D. Mortimer , Practical Laboratory Andrology (Oxford University Press, 1994).

[52] L. Boeri , P. Capogrosso , E. Ventimiglia , et al., “High‐Risk Human Papillomavirus in Semen Is Associated With Poor Sperm Progressive Motility and a High Sperm DNA Fragmentation Index in Infertile Men,” Human Reproduction 34 (2019): 209. 30517657 10.1093/humrep/dey348

[53] S. T. Mortimer , “A Critical Review of the Physiological Importance and Analysis of Sperm Movement in Mammals,” Human Reproduction Update 3 (1997): 403. 9528908 10.1093/humupd/3.5.403

[54] S. Berntsen , A. Zedeler , B. Nøhr , et al., “IVF Versus ICSI in Patients Without Severe Male Factor Infertility: A Randomized Clinical Trial,” Nature Medicine 31 (2025): 1939. 10.1038/s41591-025-03621-x PMC12176614 40217077

[55] V. Valiuškaitė , V. Raudonis , R. Maskeliūnas , R. Damaševičius , and T. Krilavičius , “Deep Learning Based Evaluation of Spermatozoid Motility for Artificial Insemination,”Sensors 21 (2021): 72. 10.3390/s21010072 PMC7795243 33374461

[56] D. Mortimer and S. T. Mortimer , “The Future of Computer‐Assisted Semen Analysis in the Evaluation of Male Infertility,” in Men’s Reproductive and Sexual Health Throughout the Lifespan: An Integrated Approach to Fertility, Sexual Function, and Vitality, ed. D. T. Carrell , A. W. Pastuszak , and J. M. Hotaling (Cambridge University Press, 2023), 165–174.

[57] C. Castellini , A. Dal Bosco , S. Ruggeri , and G. Collodel , “What Is the Best Frame Rate for Evaluation of Sperm Motility in Different Species by Computer‐Assisted Sperm Analysis?,” Fertility and Sterility 96 (2011): 24. 21723441 10.1016/j.fertnstert.2011.04.096

[58] S. T. Mortimer and M. A. Swan , “Effect of Image Sampling Frequency on Established and Smoothing‐Independent Kinematic Values of Capacitating Human Spermatozoa,” Human Reproduction 14 (1999): 997. 10221233 10.1093/humrep/14.4.997

