# Supplementary material for: An Artificial Intelligence‐Based Computer Vision Model for Human Sperm Concentration, Motility, and Kinematics Analysis
Source: Smart Med. 2026 Jan 9;5(1):e70026. doi: 10.1002/smmd.70026 (PMC12794671; doi:10.1002/smmd.70026)
Supplement: Supplementary file 1 — Supporting Information S1 [file SMMD-5-e70026-s001.docx]

**An artificial intelligence-based computer vision model for human sperm concentration, motility, and kinematics analysis**

Sahar Shahali^1^, David Mortimer^2^, Moira K. O’Bryan^3^, Robert McLachlan^4,5^, Deirdre Zander-Fox^4,6,7^, Klaus Ackermann^8^, Gulfam Ahmad^9^, Adrian Neild^1^, Reza Nosrati^1*^

*^1^ Department of Mechanical and Aerospace Engineering, Monash University, Clayton, Victoria 3800, Australia*

*^2^ Oozoa Biomedical Inc, West Vancouver, British Columbia, Canada*

*^3^ School of BioSciences and Bio21 Molecular Science and Biotechnology Institute, University of Melbourne, Parkville, Victoria 3010, Australia*

*^4^ Monash IVF Group, Cremorne, Victoria 3121, Australia*

*^5^ Clinical Andrology, Hudson Institute of Medical Research, Monash University*

*^6^ Biomedical Discovery Institute, Monash University, Clayton 3168, Australia*

*^7^ Department of Biomedicine, University of Adelaide, Adelaide 5000, Australia*

*^8^ SoDa Labs and Department of Econometrics and Business Statistics, Monash Business School, Clayton, Victoria 3800, Australia*

*^9^ Andrology, Royal Children’s Hospital, Melbourne, Victoria*

*Corresponding Author:

Department of Mechanical and Aerospace Engineering, Monash University, Clayton, Victoria 3800, Australia

Email: [Reza.Nosrati@monash.edu](mailto:Reza.Nosrati@monash.edu) (R.N.)

Phone numbers: +61 3 990 53627

**Supplementary Figures**


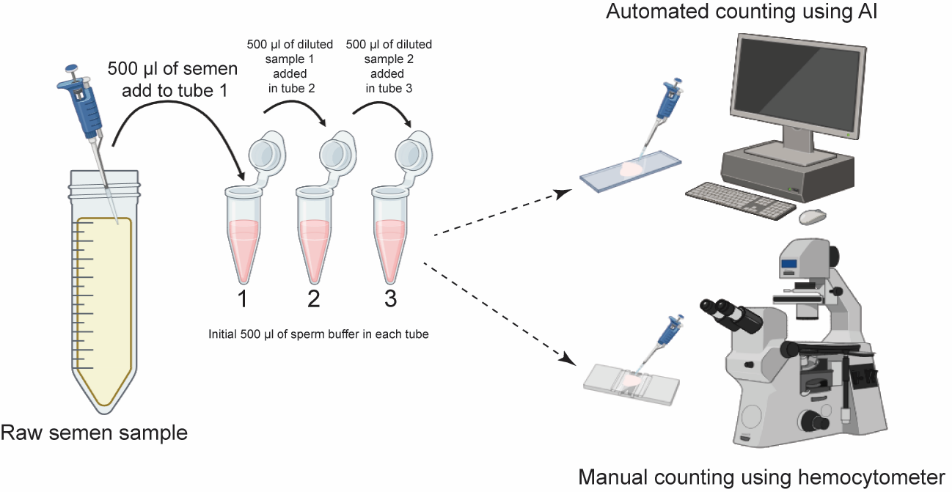


**Supplementary Figure 1. Calibration curve for sperm concentration analysis.** To generate a range of known sperm concentrations, fresh donor semen samples were serially diluted at ratios of 1-in-2, 1-in-4, 1-in-6, 1-in-8, and 1-in-10. Sperm concentration for each dilution was measured using manual counting with a hemocytometer and compared with concentration estimates obtained from the computer vision tracking model. The calibration curve was established by correlating the hemocytometer-based concentration values with the number of spermatozoa tracked in the first 10 frames of the corresponding video.


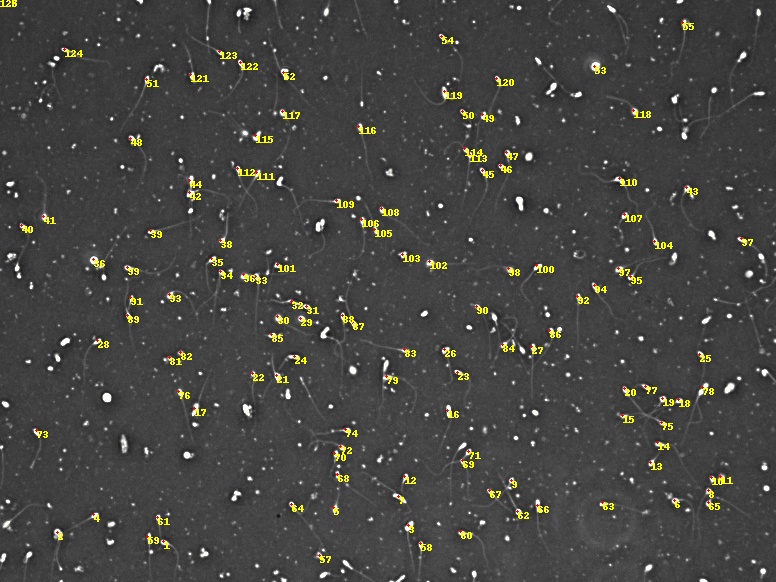


**Supplementary Figure 2. Mapping of sperm cell IDs in the first frame of the video for consistent tracking across methods**. Each sperm cell was assigned a unique ID number, shown in the first frame to ensure that the same cells were tracked and analyzed across the methods.


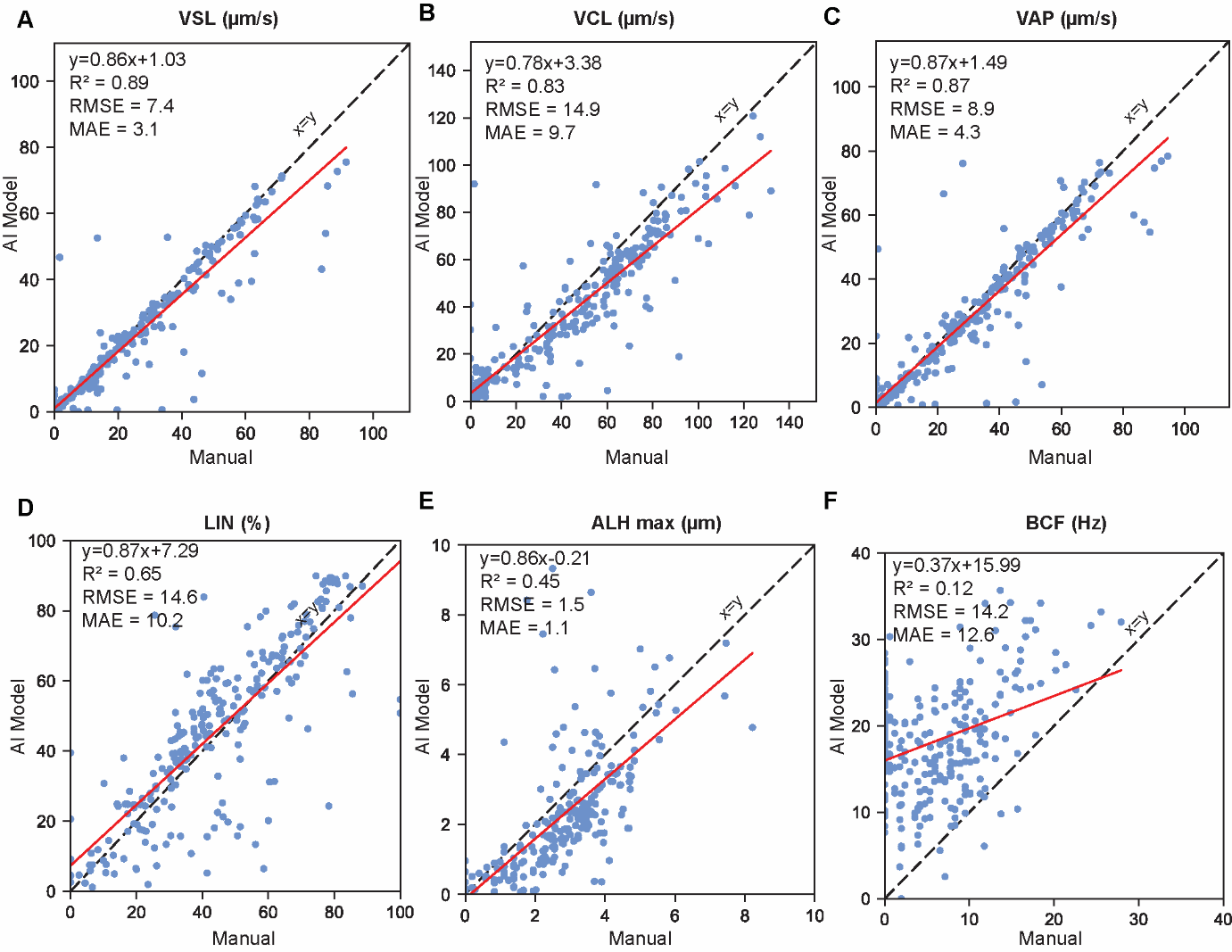


**Supplementary Figure 3.** **Single-cell linear regression analysis comparing motility parameters between the AI-model and manual tracking. (A)** straight line velocity (VSL). **(B)** curvilinear velocity (VCL), **(C)** average path velocity (VAP), **(D)** linearity (LIN), **(E)** maximum amplitude of lateral head displacement (ALH_max_), **(F)** beat cross frequency (BCF). Each point represents an individual spermatozoon. The solid red line indicates the best-fit linear regression.


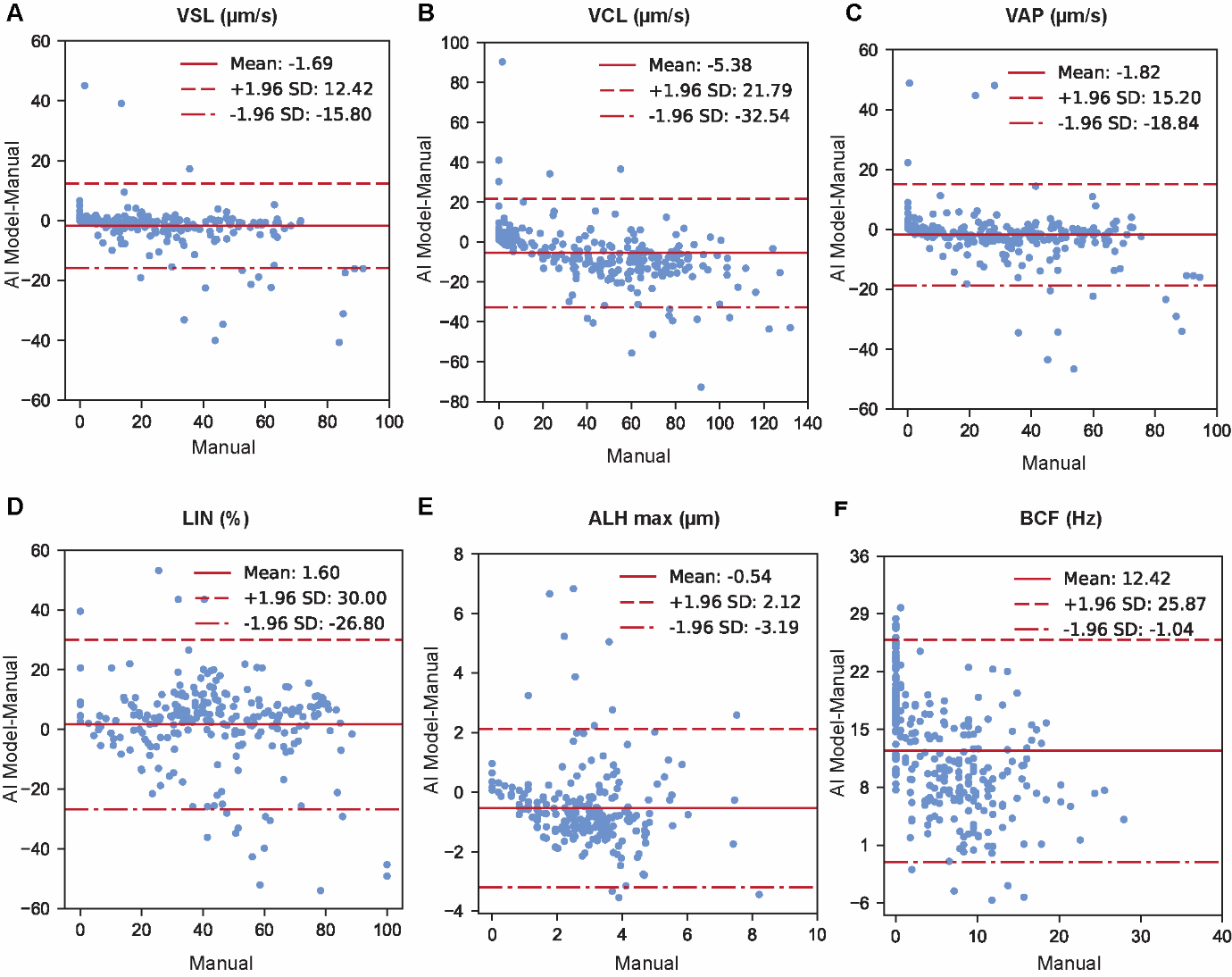


**Supplementary Figure 4. Comparison of single cell-level motility parameters between the AI-model and manual tracking using Bland and Altman plots.** **(A)** VSL, **(B)** VCL, **(C)** VAP, **(D)** LIN, **(E)** ALH_max_, and **(F)** BCF (abbreviations as defined for Supplementary Figure 3). The x-axis shows the values obtained by the reference manual method, while the y-axis shows the difference between values from the two methods. The solid red line indicates the mean difference, and the dashed red lines represent the 95% range (i.e., mean ±1.96 SD for n»120).


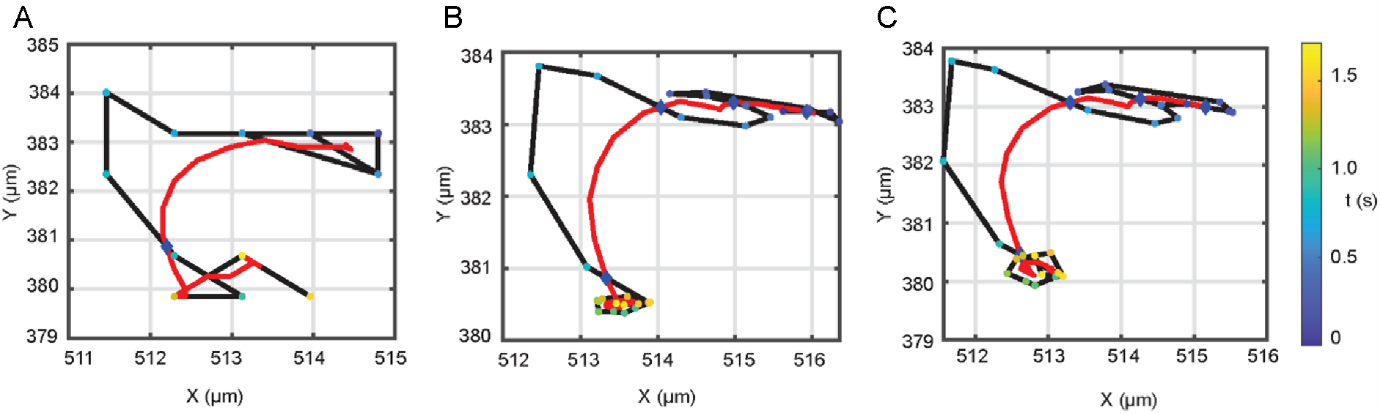


**Supplementary Figure 5. Comparison of representative swimming trajectories across the three methods.** The average path (red) and instantaneous swimming trajectory (black) are shown for **(A)** manual tracking, **(B)** the AI model, and **(C)** CASA, yielding BCF values of 7 Hz, 14 Hz, and 113 Hz, respectively. The number of intersections between the average path and the actual trajectory per unit time corresponds to the BCF. The color legend indicates time in seconds.


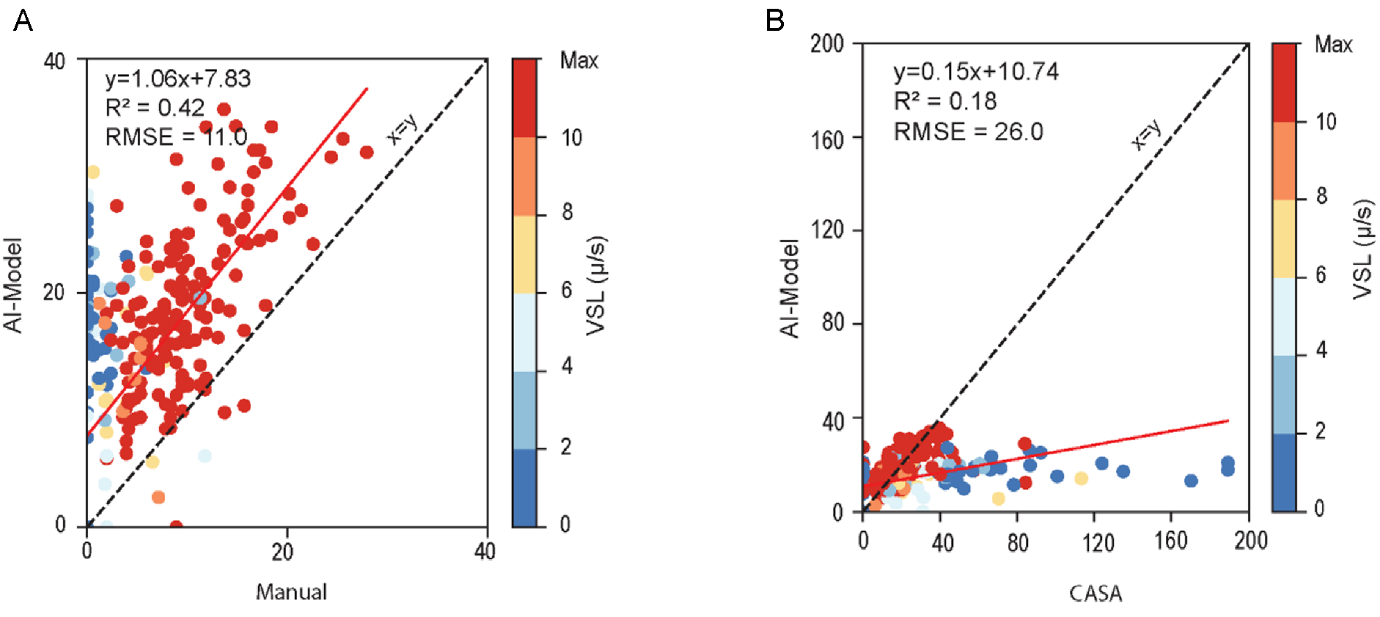


**Supplementary Figure 6. Comparison of single-cell BCF values across the three methods. (A)** BCF values from the AI-based model plotted against manual tracking. **(B)** BCF values from CASA compared with manual tracking. Data points are color-coded based on sperm VSL, and the solid red line represents the best-fit linear regression.


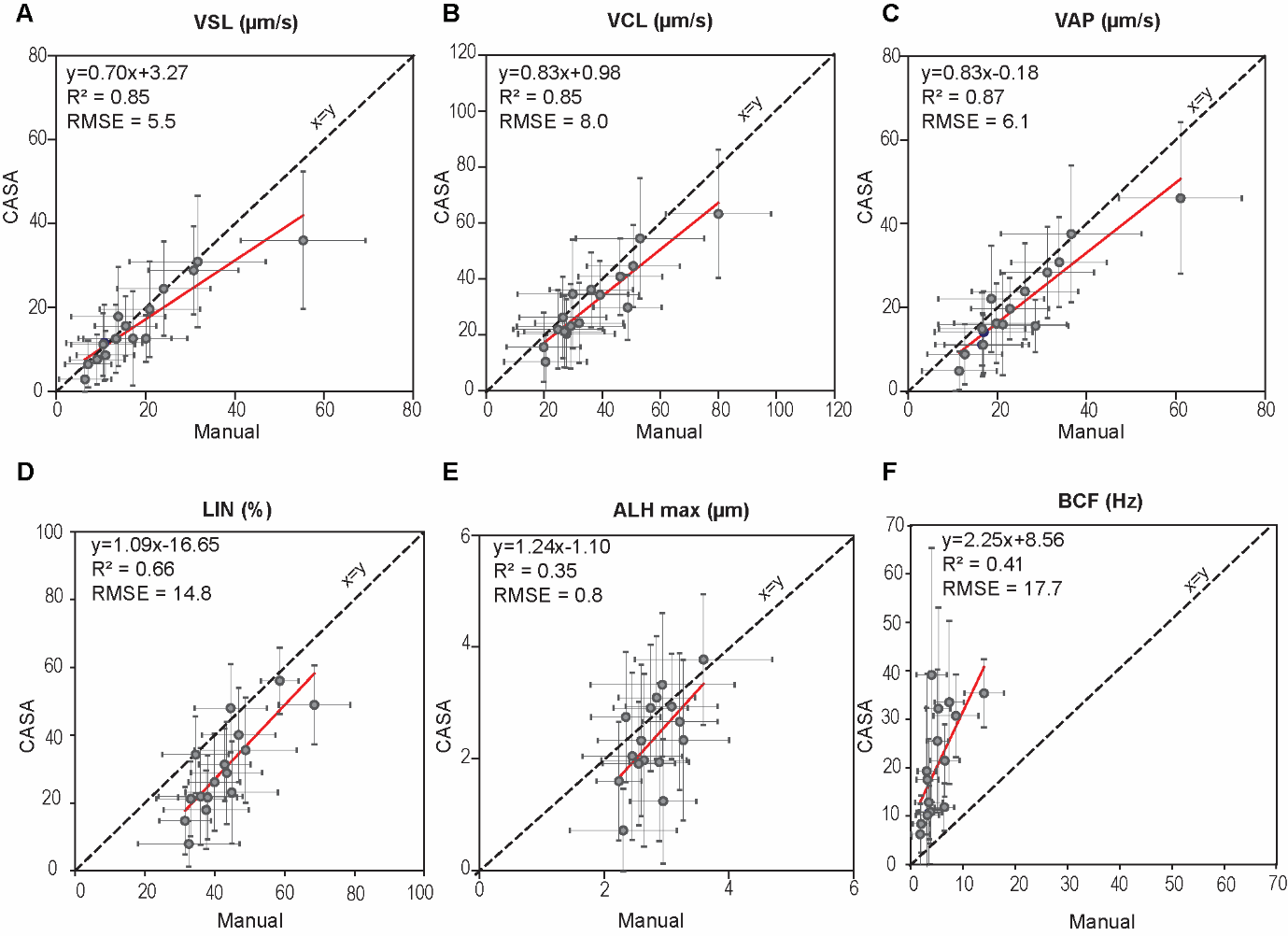


**Supplementary Figure 7. Linear regression analysis comparing between CASA and manual tracking motility parameters. (A)** VSL, **(B)** VCL, **(C)** VAP, **(D)** LIN, **(E)** ALH_max_, and **(F)** BCF (abbreviations as defined for Supplementary Figure 3). Each point represents the average motility parameter per sample (n=16), presented as mean ± SD calculated from 20 spermatozoa per sample in the manual tracking dataset. The solid red line indicates the best-fit linear regression.


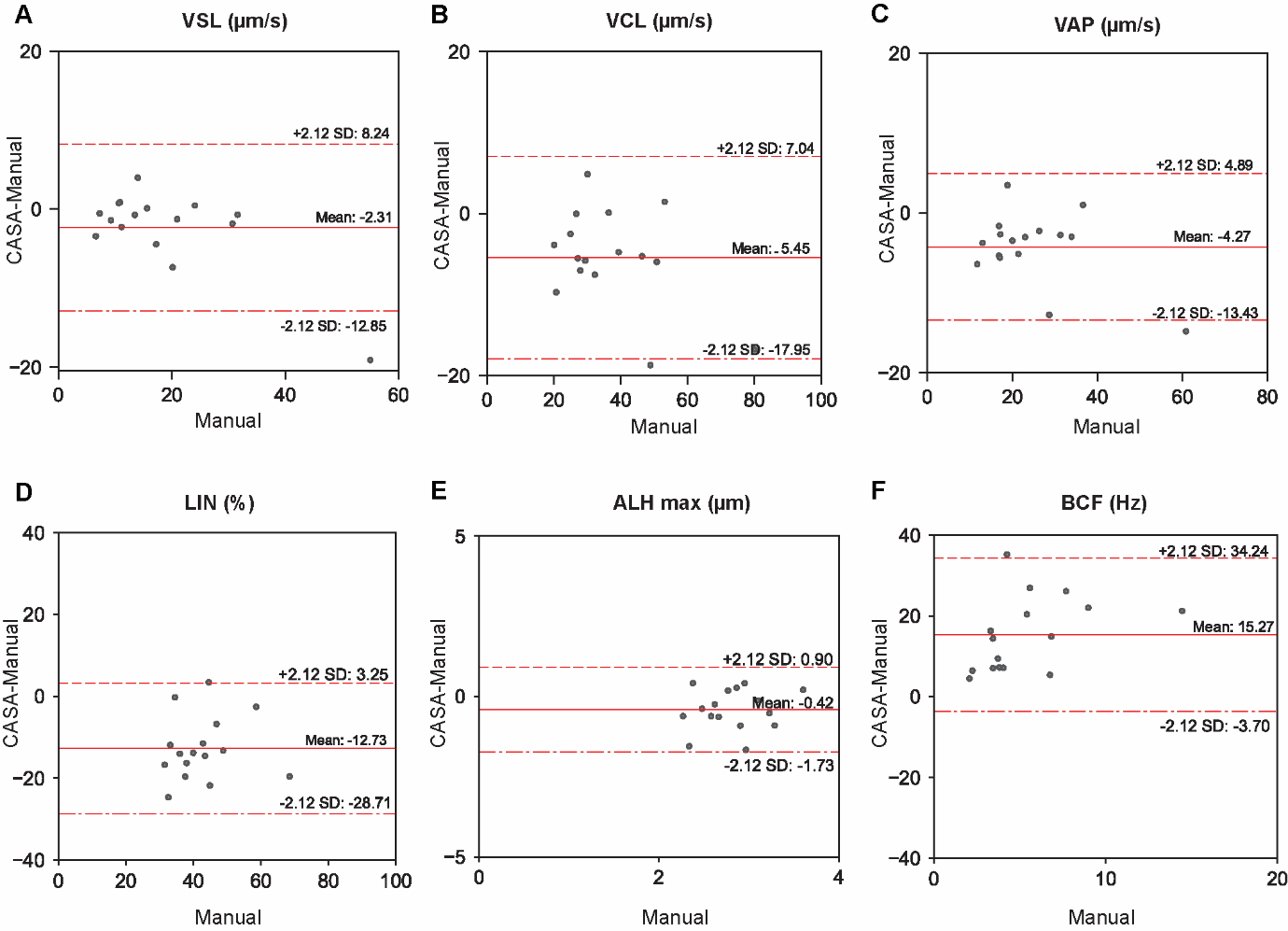


**Supplementary Figure 8. Comparison of population-level motility parameters between CASA and manual tracking using Bland and Altman plots.** **(A)** VSL, **(B)** VCL, **(C)** VAP, **(D)** LIN, **(E)** ALH_max_, and **(F)** BCF (abbreviations as defined for Supplementary Figure 3). The x-axis shows the values obtained by the reference manual method, while the y-axis shows the difference between values from the two methods. The solid red line indicates the mean difference, and the dashed red lines represent the 95% range (i.e., mean ±2.12 SD for n=16).


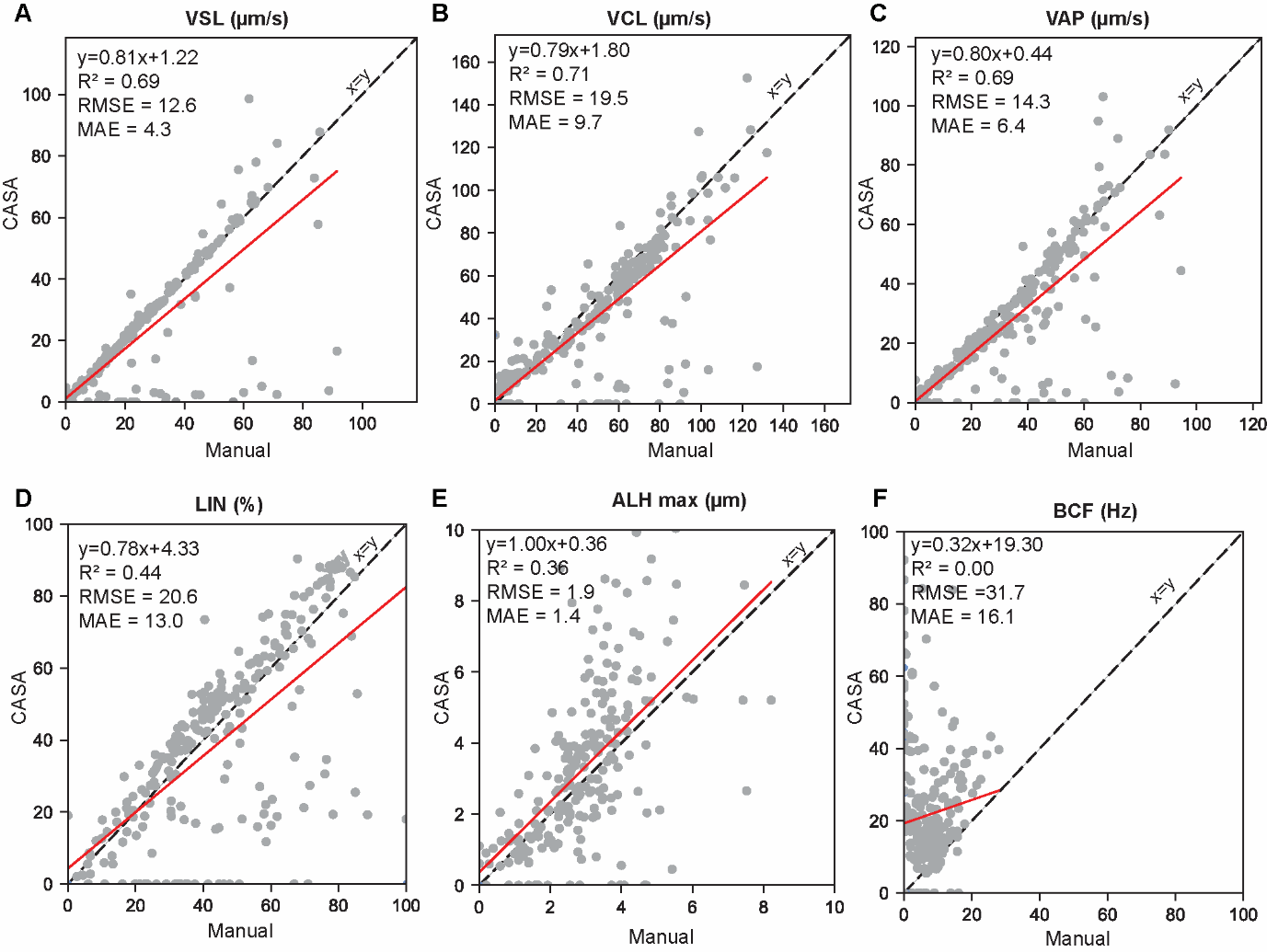


**Supplementary Figure 9. Single-cell linear regression analysis comparing motility parameters between CASA and manual tracking. (A)** VSL, **(B)** VCL, **(C)** VAP, **(D)** LIN, **(E)** ALH_max_, and **(F)** BCF. Each point represents an individual spermatozoon. The solid red line indicates the best-fit linear regression.


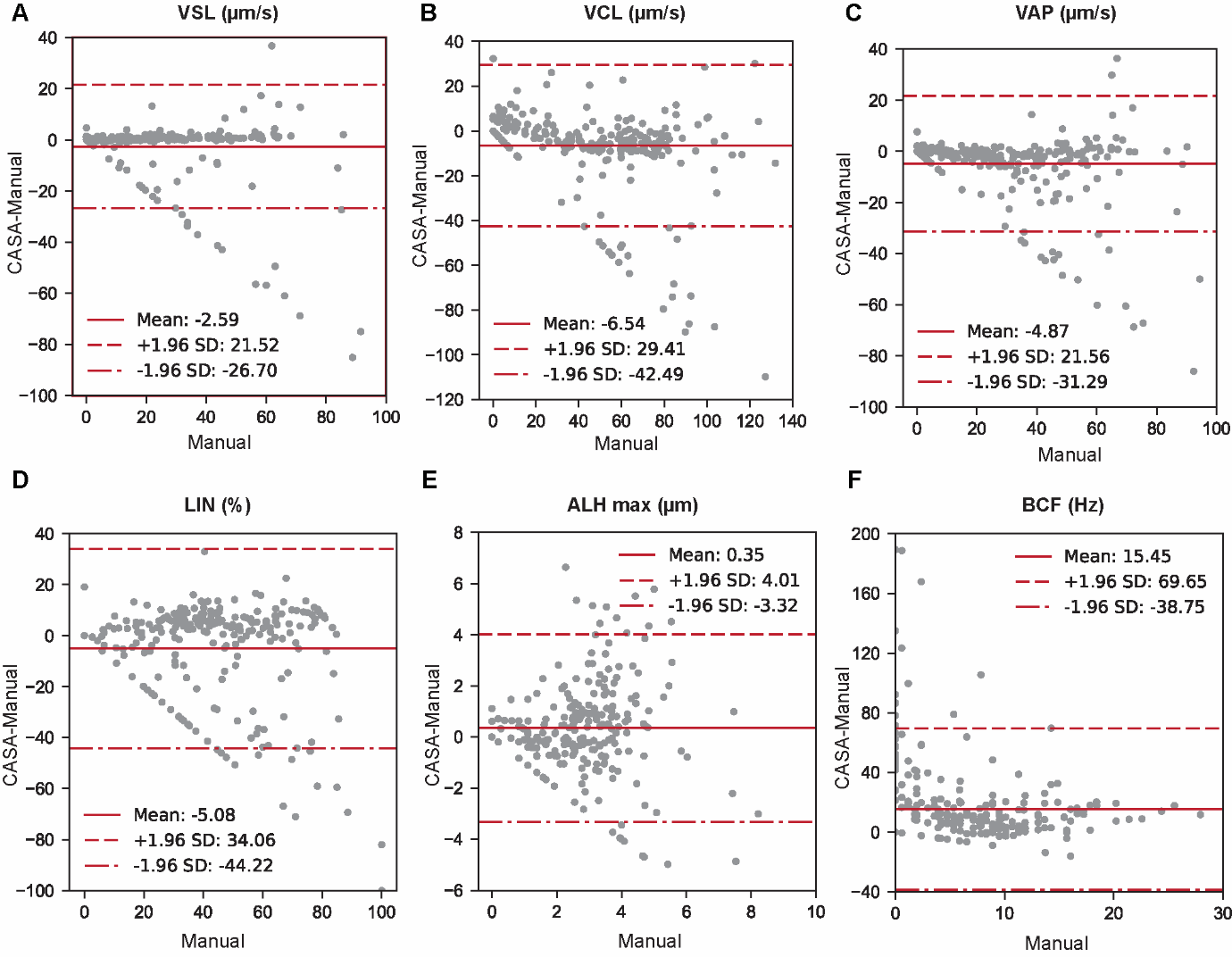


**Supplementary Figure 10. Comparison of single cell-level motility parameters between CASA and manual tracking using Bland and Altman plots.** **(A)** VSL, **(B)** VCL, **(C)** VAP, **(D)** LIN, **(E)** ALH_max_, and **(F)** BCF (abbreviations as defined for Supplementary Figure 3). The x-axis shows the values obtained by the reference manual method, while the y-axis shows the difference between values from the two methods. The solid red line indicates the mean difference, and the dashed red lines represent the 95% range (i.e., mean ±1.96 SD).


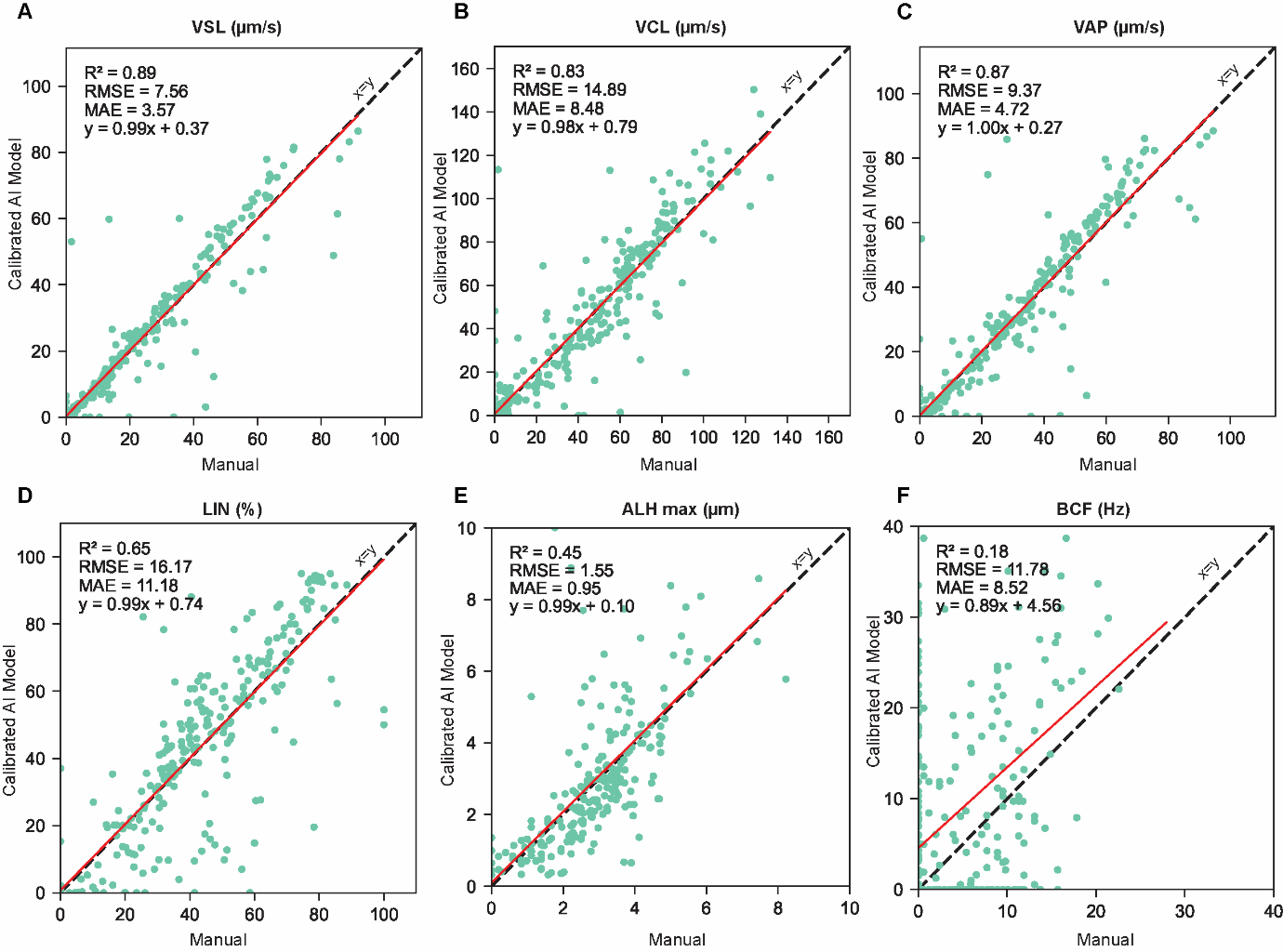


**Supplementary Figure 11. Single-cell linear regression analysis comparing motility parameters between the calibrated AI-model and manual tracking. (A)** VSL. **(B)** VCL. **(C)** VAP. **(D)** LIN. **(E)** ALH_max_. **(F)** BCF (abbreviations as defined for Supplementary Figure 3). Each point represents an individual spermatozoon. The solid red line indicates the best-fit linear regression.


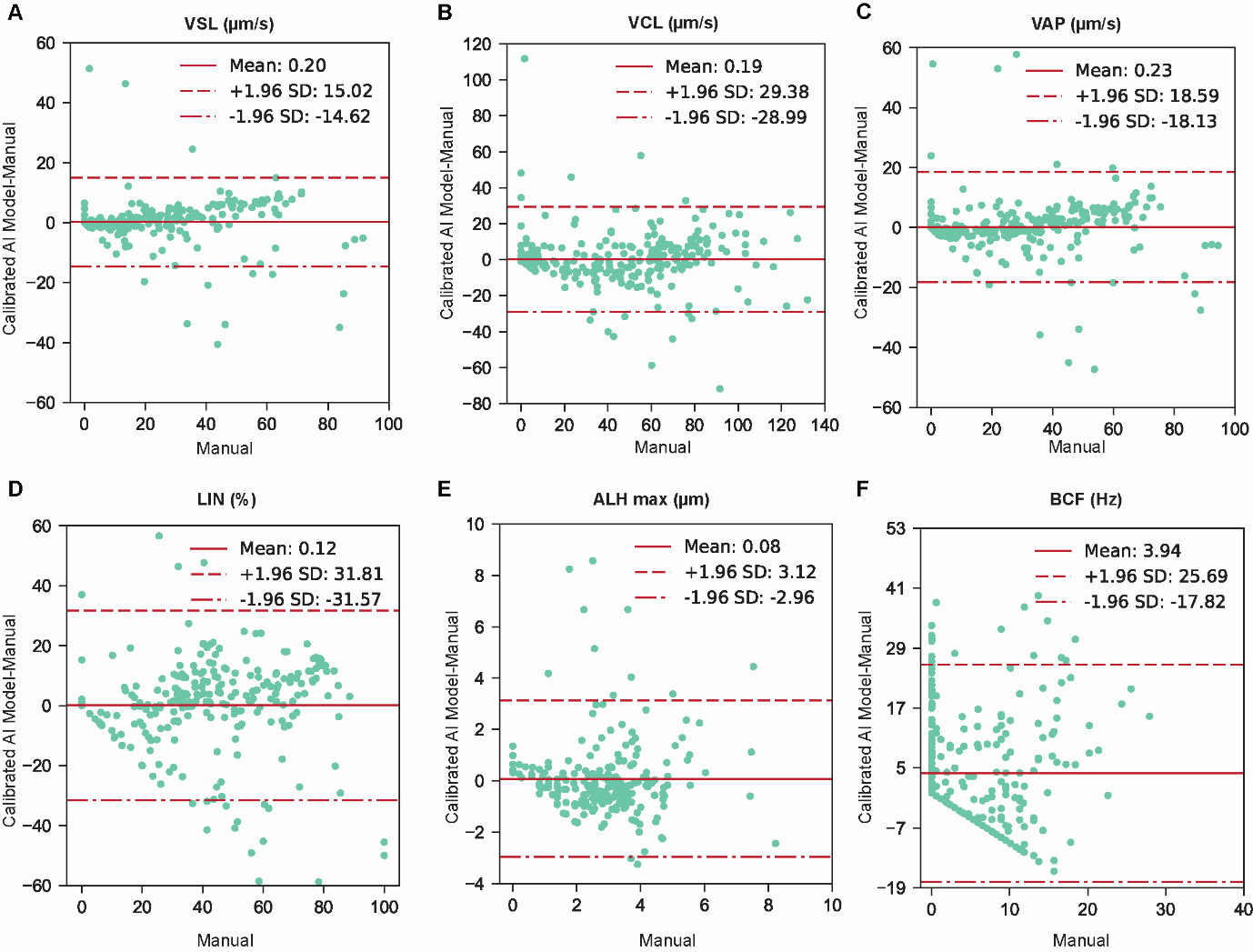


**Supplementary Figure 12. Comparison of single cell-level motility parameters between the calibrated AI-model and manual tracking using Bland and Altman plots.** **(A)** VSL, **(B)** VCL, **(C)** VAP, **(D)** LIN, **(E)** ALH_max_, and **(F)** BCF (abbreviations as defined for Supplementary Figure 3). The x-axis shows the values obtained by the reference manual method, while the y-axis shows the difference between values from the two methods. The solid red line indicates the mean difference, and the dashed red lines represent the 95% range (i.e., mean ±1.96 SD).


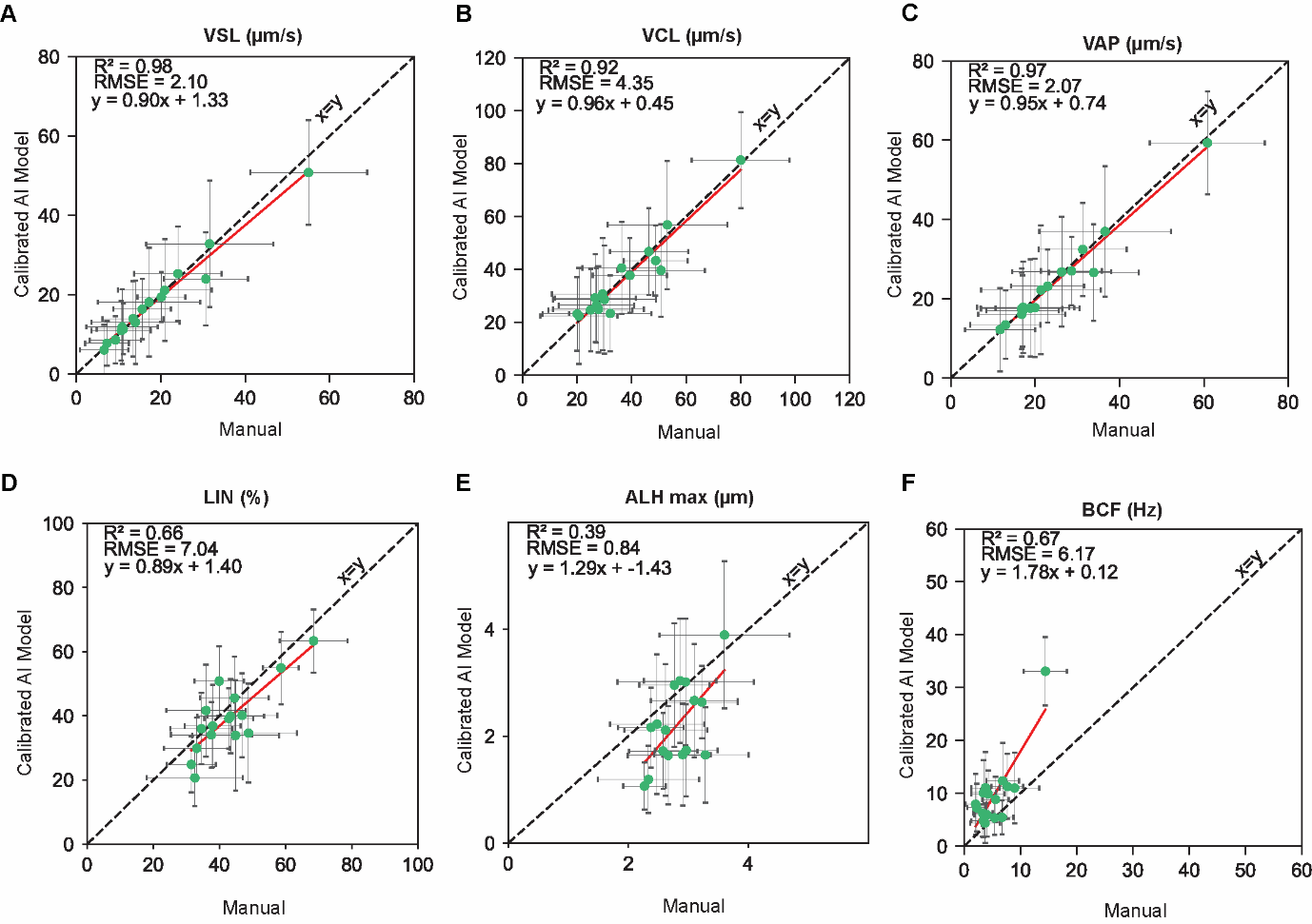


**Supplementary Figure 13. Linear regression analysis comparing between calibrated AI model and manual tracking motility parameters. (A)** VSL, **(B)** VCL, **(C)** VAP, **(D)** LIN, **(E)** ALH_max_, and **(F)** BCF (abbreviations as defined for Supplementary Figure 3). Each point represents the average motility parameter per sample (n=16), presented as mean ± SD calculated from 20 spermatozoa per sample in the manual tracking dataset. The solid red line indicates the best-fit linear regression.


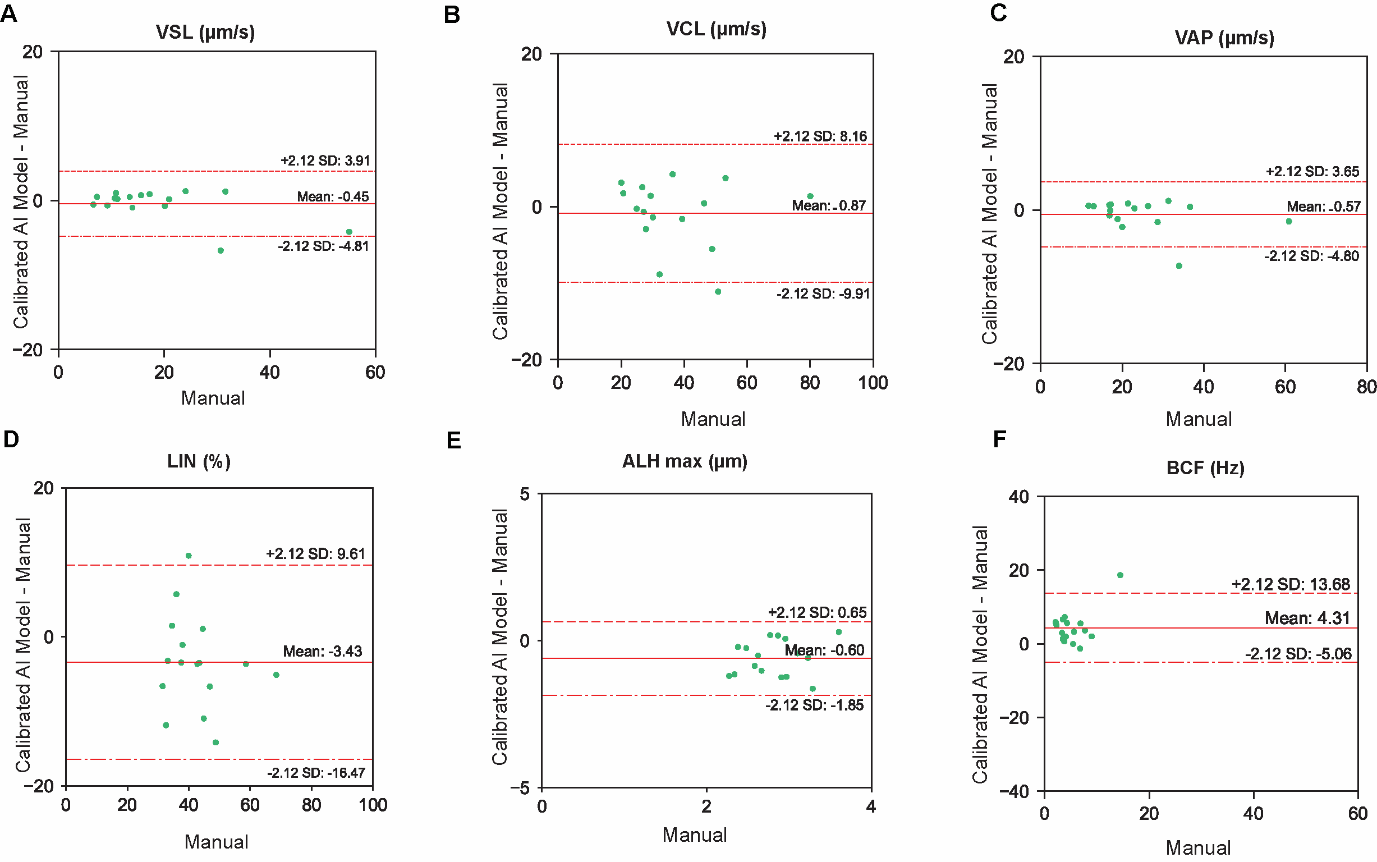


**Supplementary Figure 14. Comparison of population-level motility parameters between calibrated AI model and manual tracking using Bland and Altman plots.** **(A)** VSL, **(B)** VCL, **(C)** VAP, **(D)** LIN, **(E)** ALH_max_, and **(F)** BCF (abbreviations as defined for Supplementary Figure 3). The x-axis shows the values obtained by the reference manual method, while the y-axis shows the difference between values from the two methods. The solid red line indicates the mean difference, and the dashed red lines represent the 95% range (i.e., mean ±2.12 SD).


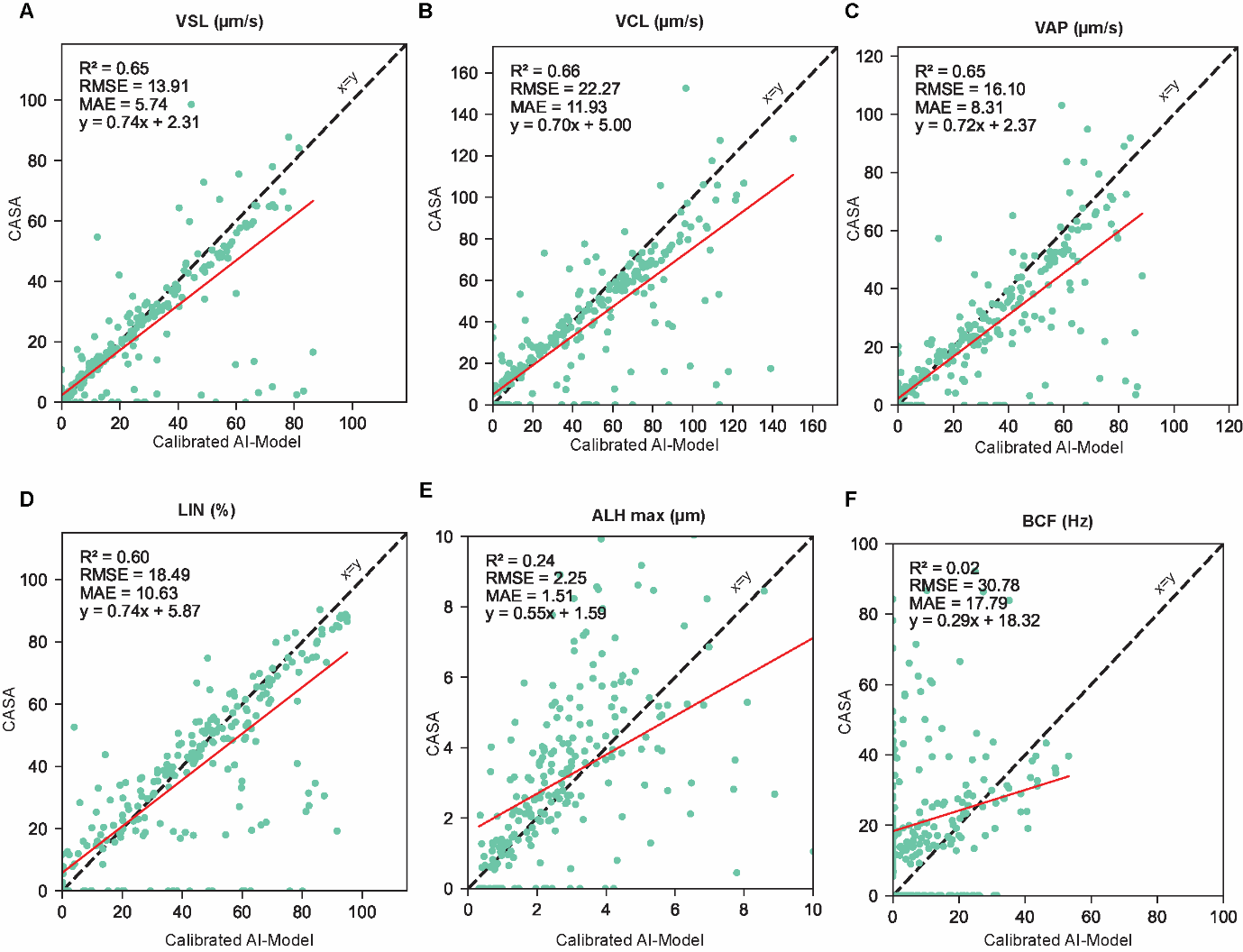


**Supplementary Figure 15. Single-cell linear regression analysis comparing motility parameters between CASA and the calibrated AI-model. (A)** VSL, **(B)** VCL, **(C)** VAP, **(D)** LIN, **(E)** ALH_max_, **(F)** BCF (abbreviations as defined for Supplementary Figure 3). Each point represents an individual spermatozoon. The solid red line indicates the best-fit linear regression.


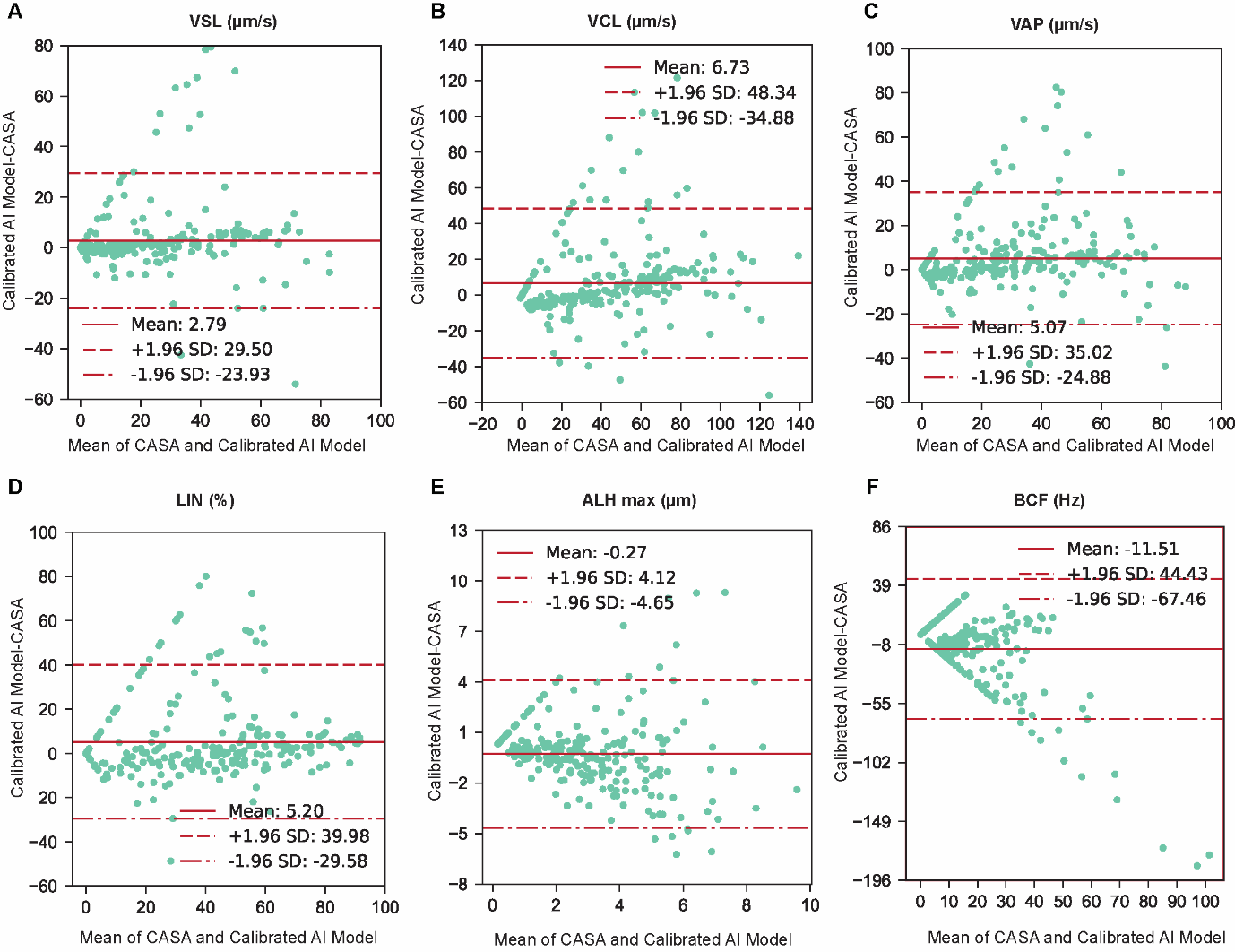


**Supplementary Figure 16. Comparison of single cell-level motility parameters between CASA and the calibrated AI-model using Bland and Altman plots.** **(A)** VSL, **(B)** VCL, **(C)** VAP, **(D)** LIN, **(E)** ALH_max_, and **(F)** BCF (abbreviations as defined for Supplementary Figure 3). The x-axis represents the mean value from the two methods, while the y-axis shows the difference between values from the two methods. The solid red line indicates the mean difference, and the dashed red lines represent the 95% range (i.e., mean ±1.96 SD).

**Supplementary Table 1.** **Sperm motility grading across methods.** The percentage of immotile and non-progressive (IM&NP), slow progressive, and rapid progressive spermatozoa for manual tracking, CASA, and the calibrated AI model, across 16 patient samples.

| **Sample No.** | **Motility grade** | **Manual tracking (%)** | **CASA**  **(%)** | **Calibrated**  **AI (%)** | **Manual vs CASA (% difference)** | **Manual vs AI (% difference)** |
| --- | --- | --- | --- | --- | --- | --- |
| 1 | *IM & NP*  *Slow progressive*  *Rapid progressive* | 50  28  22 | 40  33  27 | 50  33  17 | 20  -18  -23 | 0  -18  23 |
| 2 | *IM & NP*  *Slow progressive*  *Rapid progressive* | 67  28  6 | 79  21  0 | 67  28  6 | -18  25  100 | 0  0  0 |
| 3 | *IM & NP*  *Slow progressive*  *Rapid progressive* | 44  38  18 | 53  20  27 | 50  25  25 | -20  47  -50 | -14  34  -39 |
| 4 | *IM & NP*  *Slow progressive*  *Rapid progressive* | 55  25  20 | 63  16  21 | 60  25  15 | -15  36  -5 | -9  0  25 |
| 5 | *IM & NP*  *Slow progressive*  *Rapid progressive* | 58  26  16 | 59  24  17 | 58  16  26 | -2  8  -6 | 0  38  -63 |
| 6 | *IM & NP*  *Slow progressive*  *Rapid progressive* | 50  31  19 | 60  27  13 | 50  31  19 | -20  13  32 | 0  0  0 |
| 7 | *IM & NP*  *Slow progressive*  *Rapid progressive* | 11  53  36 | 35  47  18 | 21  37  42 | -218  11  50 | -91  30  -17 |
| 8 | *IM & NP*  *Slow progressive*  *Rapid progressive* | 32  45  23 | 32  45  23 | 32  42  26 | 0  0  0 | 0  7  -13 |
| 9 | *IM & NP*  *Slow progressive*  *Rapid progressive* | 21  16  63 | 25  19  56 | 37  21  42 | -19  -19  11 | -76  -31  33 |
| 10 | *IM & NP*  *Slow progressive*  *Rapid progressive* | 24  35  41 | 23  37  40 | 26  34  40 | 4  -6  2 | -8  3  2 |
| 11 | *IM & NP*  *Slow progressive*  *Rapid progressive* | 5  16  79 | 21  26  53 | 5  16  79 | -320  -63  33 | 0  0  0 |
| 12 | *IM & NP*  *Slow progressive*  *Rapid progressive* | 40  7  53 | 40  13  47 | 40  7  53 | 0  -86  11 | 0  0  0 |
| 13 | *IM & NP*  *Slow progressive*  *Rapid progressive* | 45  20  35 | 50  15  35 | 50  15  35 | -11  25  0 | -11  25  0 |
| 14 | *IM & NP*  *Slow progressive*  *Rapid progressive* | 61  11  28 | 77  0  23 | 67  6  27 | -26  100  18 | -10  45  4 |
| 15 | *IM & NP*  *Slow progressive*  *Rapid progressive* | 67  22  11 | 73  13  14 | 67  22  11 | -9  41  -27 | 0  0  0 |
| 16 | *IM & NP*  *Slow progressive*  *Rapid progressive* | 53  32  15 | 50  33  17 | 53  32  15 | 6  -3  -13 | 0  0  0 |

**Supplementary Table 2.** Comparative summary of recent AI sperm tracking and motility analysis methods

| **Reference** | **Dataset** | **Model architecture** | **Performance evaluation** | **Ground truth** |
| --- | --- | --- | --- | --- |
| Mohammadi, et al. 2020 | 36 videos of 25 frames at 50 fps | RetinaNet detection with CSR-DCF tracking | Detection precision 99.1%; tracking F1 96.6% | Manual annotations |
| Ottl, et al. 2022 | VISEM (85 videos, 50 fps) | Linear Support Vector regression | MAE = 7.31 for sample-level motility-grade prediction | Manual visual inspection |
| Haugen, et al. 2023 | 65 videos of 5–10 s at 30 fps | ResNet-50 | MAE < 0.1 for sample-level motility-grade prediction | Manual visual inspection |
| Valiuškaitė, *et al*. 2021 | VISEM (85 videos, 50 fps) | Region-Based Convolutional Neural Networks (R-CNN) | Head-detection accuracy 91.7%; vitality prediction MAE 2.92 | Manual annotations |
| **Current Study** | 110 videos at 60 fps for 1.7 s | Optical flow tracking and regression analysis | \| Strong agreement in single-cell kinematics and WHO grading \| \| --- \| | Manual frame-by-frame tracking |
